# DNA satellite and chromatin organization at mouse centromeres and pericentromeres

**DOI:** 10.1186/s13059-024-03184-z

**Published:** 2024-02-20

**Authors:** Jenika Packiaraj, Jitendra Thakur

**Affiliations:** Department of Biology, Emory University, 1510 Clifton Rd, Atlanta, GA 30322 USA

**Keywords:** CENP-A, H3K9me3, Constitutive heterochromatin, Long-read sequencing, Transposable elements, Repetitive DNA

## Abstract

**Background:**

Centromeres are essential for faithful chromosome segregation during mitosis and meiosis. However, the organization of satellite DNA and chromatin at mouse centromeres and pericentromeres is poorly understood due to the challenges of assembling repetitive genomic regions.

**Results:**

Using recently available PacBio long-read sequencing data from the C57BL/6 strain, we find that contrary to the previous reports of their homogeneous nature, both centromeric minor satellites and pericentromeric major satellites exhibit a high degree of variation in sequence and organization within and between arrays. While most arrays are continuous, a significant fraction is interspersed with non-satellite sequences, including transposable elements. Using chromatin immunoprecipitation sequencing (ChIP-seq), we find that the occupancy of CENP-A and H3K9me3 chromatin at centromeric and pericentric regions, respectively, is associated with increased sequence enrichment and homogeneity at these regions. The transposable elements at centromeric regions are not part of functional centromeres as they lack significant CENP-A enrichment. Furthermore, both CENP-A and H3K9me3 nucleosomes occupy minor and major satellites spanning centromeric-pericentric junctions and a low yet significant amount of CENP-A spreads locally at centromere junctions on both pericentric and telocentric sides. Finally, while H3K9me3 nucleosomes display a well-phased organization on major satellite arrays, CENP-A nucleosomes on minor satellite arrays are poorly phased. Interestingly, the homogeneous class of major satellites also phase CENP-A and H3K27me3 nucleosomes, indicating that the nucleosome phasing is an inherent property of homogeneous major satellites.

**Conclusions:**

Our findings reveal that mouse centromeres and pericentromeres display a high diversity in satellite sequence, organization, and chromatin structure.

**Supplementary Information:**

The online version contains supplementary material available at 10.1186/s13059-024-03184-z.

## Background

Centromeres are the chromosomal sites where spindle fibers attach via the kinetochore to allow chromosome segregation during cell division. Defects in centromere function can cause chromosome missegregation and aneuploidy, which are linked to cancers, miscarriages, and genetic disorders [1–4]. Centromeres are characterized by specialized nucleosomes composed of Centromere-Protein A (CENP-A), which replaces canonical histone H3 at centromeric chromatin [5, 6]. CENP-A chromatin acts as the foundation for the assembly of kinetochore components. In mammals, CENP-A is assembled on long arrays of tandem DNA repeats called satellites [7]. Human centromeres comprise α-satellite (171 bp monomer) arrays, some of which are further organized as higher-order repeat (HOR) structures [8–10]. A highly homogeneous α-satellite core forms the functional centromere, which is flanked by more divergent α-satellite monomers [8–11]. Due to the lack of conserved centromeric sequences, CENP-A chromatin is considered the epigenetic mark of centromeres. This is further supported by the formation of functional ectopic centromeres, called neocentromeres, at locations lacking satellite sequences [12, 13]. CENP-A chromatin has been extensively studied through in vitro reconstitution, demonstrating the presence of octameric, hexameric, and tetrameric CENP-A nucleosomes in various eukaryotes [14–19]. In vivo studies using tagged CENP-A pulldown have also revealed the existence of CENP-A dimers within nucleosomes. However, the centromeric chromatin organization on satellite arrays in vivo remains poorly understood. Centromeres of *Saccharomyces cerevisiae* are defined by genetically specific sequence motifs (CDE1, CEDII, and CDEIII elements) and contain one CENP-A nucleosome precisely positioned on CDE elements [17]. In contrast, epigenetic centromeres in *Schizosaccharomyces pombe* do not display discernible CENP-A positioning and phasing [20]. In Rice, centromeric 155 bp CentO repeats exhibit strong positioning and phasing of CENP-A nucleosomes [21]. In humans, dimeric α-satellites, characterized by high homogeneity, exhibit CENP-A positioning, while the more divergent HORs lack this characteristic organization [22]. At majority of homogeneous α-satellite arrays, a 340 bp α-satellite dimeric unit is occupied by two CENP-A particles bridged by a CENP-B, CENP-C, and CENP-T containing linker [22, 23]. Furthermore, sequence variations across different α-satellite dimers within a given array on a given chromosome corresponded to variations in CENP-A chromatin profiles, suggesting a sequence-dependent assembly of centromeric chromatin [24].

In most plants and animals, centromeres are flanked by pericentric regions that are also highly repetitive [7]. Pericentromeric regions assemble distinct constitutive heterochromatin in which histone H3 is trimethylated at its lysine 9 residue (H3K9me3) [25–27]. Pericentric heterochromatin binds to cohesin, which is required for proper chromosome segregation by preventing sister chromatid separation before anaphase [28, 29]. Unlike centromeres, where a single type of satellite array is present, human pericentric satellites have undergone extensive divergence, resulting in distinct satellite families. These include HSATI (comprising 17-bp and 25-bp repeat units), HSAT II (containing 10–80-bp repeat units), HSATIII (comprising 5-bp and 10-bp repeat units), beta satellites, and gamma satellites [7, 9]. Each HSAT family harbors unique DNA sequences, often displaying copy number variations in different cell lines. Additionally, human pericentric regions, particularly HSATI arrays, exhibit substantial structural rearrangements, including inversions. Furthermore, while centromeric α-satellites are mostly devoid of transposable elements (TEs), HSATI and HSATII repeat units are interspersed with ancient inactive TEs [9]. The human pericentromeric regions also contain frequent transposable elements (TEs) [9, 30]. TEs are also found at the functional centromeres of other eukaryotes, including *Drosophila* [31]. Furthermore, centromeric and pericentric satellite sequences and organization can vary greatly, even between chromosomes within the same individual, as seen in humans [9, 32–35].

Despite their essential role in chromosome segregation, sequencing and assembling centromeres and pericentric regions have been challenging due to the highly repetitive nature of DNA at these regions [36]. As a result, centromeres and other repetitive elements have been omitted or only partially annotated in genome assemblies. The lack of centromere and pericentromere assemblies has thus limited studies of CENP-A and H3K9me3 chromatin structure using genomics-based chromatin profiling methods. However, recent advances in high-fidelity long-read sequencing (LRS) have opened the possibility for further in-depth analysis of centromere organization and chromatin structure [37, 38]. In addition, the LRS technologies have led to the development of the Telomere-to-Telomere (T2T) gapless human genome assembly, which has allowed the characterization of centromeric and pericentromeric arrays in humans [9, 39]. In contrast, a comprehensive characterization of mouse centromeric satellite arrays has begun only recently. Mouse centromeres are telocentric and are defined by arrays of minor satellites (120 bp monomer) [40, 41]. Minor satellite (MiSat) arrays are flanked by TeLoCentric (TLC) satellite arrays on the telomeric side. TLC satellites are 145–146 bp repeats found near telomeres in most *Mus musculus* species that share 60–70% sequence homology with minor satellites [42]. MiSat arrays are flanked by pericentromeric major satellites (MaSat) (234 bp monomer) on the chromosome arm side [43]. MiSats are associated with the centromere proteins such as CENP-A, CENP-B, and CENP-C, while MaSats are associated with heterochromatin protein 1α (HP1) [44–46]. Both MaSat and to a lesser extent, MiSat, have been shown to contain H3K9me3 [44, 46]. H3K9me3 is shown to exhibit a specific repeating dinucleosomal configuration on major satellites, while minor satellites display simple mononucleosomal H3K9me3 configuration [44]. Unlike human α-satellites, which share 60–100% sequence similarity, mouse MiSat and MaSat arrays were previously thought to be highly homogeneous with few sequence variations within an array and between chromosomes [42, 47]. Analyses of whole genome Illumina short sequencing reads from the mouse reverence C57BL/6 strain have revealed that MiSats exhibit 5.9% sequence variations both locally and globally within a genome and are polymorphic at the 17-bp CENP-B box motif that binds Centromere Protein B (CENP-B) [45, 48]. A small fraction of MiSats also differ in the monomeric unit length [48]. These findings suggest detectable sequence variations across MiSats within a mouse genome while still indicating a substantial degree of sequence homogeneity compared to human α-satellites. Further studies have identified a considerable sequence heterogeneity and copy number of variations of MiSat across different mouse populations and strains [45, 49]. The variations include sites of high sequence variation at the CENP-B box motif [49]. Sequence variations across MaSat arrays remain poorly understood. More importantly, it remains unclear how MiSats and MaSats are arranged across long regions at centromeres and pericentromeres.

In this study, we investigated the sequence and organization of MiSat and MaSat arrays and associated chromatin in *Mus musculus* reference strain C57BL/6. First, we identified long satellite arrays by analyzing publicly available PacBio LRS data [50]. We uncovered a high degree of both global and local sequence variations within centromeric, pericentromeric, and centromere-telomere junction satellites in the C57BL/6 genome. We found up to 30%, 36.3%, and 31.6% global variations among the total pool of monomeric units for MiSat, MaSat, and TLC satellites, respectively. At the local level on satellite arrays, we found specific patterns of variations, where some arrays exhibited high homogeneity with up to 92–99.6% sequence identity among repeat units, while others showed high divergence with sequence identity among repeat units as low as 63.3%. For MiSats, sequence variations were notably concentrated at specific positions, including those within the CENP-B box sequence. Conversely, MaSat and TLC Sat units displayed sequence variations distributed throughout their entire length. In addition to nucleotide variations, we found variations in satellite organization within and across MiSat and MaSat arrays. Although the majority of satellites were present as continuous arrays, we also detected TEs interspersed with satellites in a significant fraction of both MiSat and MaSat arrays. Subsequently, we analyzed the organization of CENP-A chromatin along with constitutive H3K9me3 and facultative H3K27me3 heterochromatin at centromeric MiSat and pericentromeric MaSat arrays by generating high-resolution Chromatin immunoprecipitation Sequencing (ChIP-seq) data for CENP-A, H3K9me3, and H3K27me3. We found that the enrichment of CENP-A and H3K9me3 at both centromeric and pericentromeric regions differs on arrays containing different satellite variants. Furthermore, TEs at centromeric regions were not bound to CENP-A, indicating their absence from the functional centromeric domains. Interestingly, MaSats flanking centromeres on the chromosome arm sides were significantly enriched for CENP-A nucleosomes. Finally, we found that while MaSat arrays contain H3K9me3 nucleosomes in a well-phased configuration, MiSat arrays contain CENP-A nucleosomes that lack a phased configuration.

## Results

### Mouse major, minor and TLC satellites exhibit high global sequence variations

**Fig. 1:**
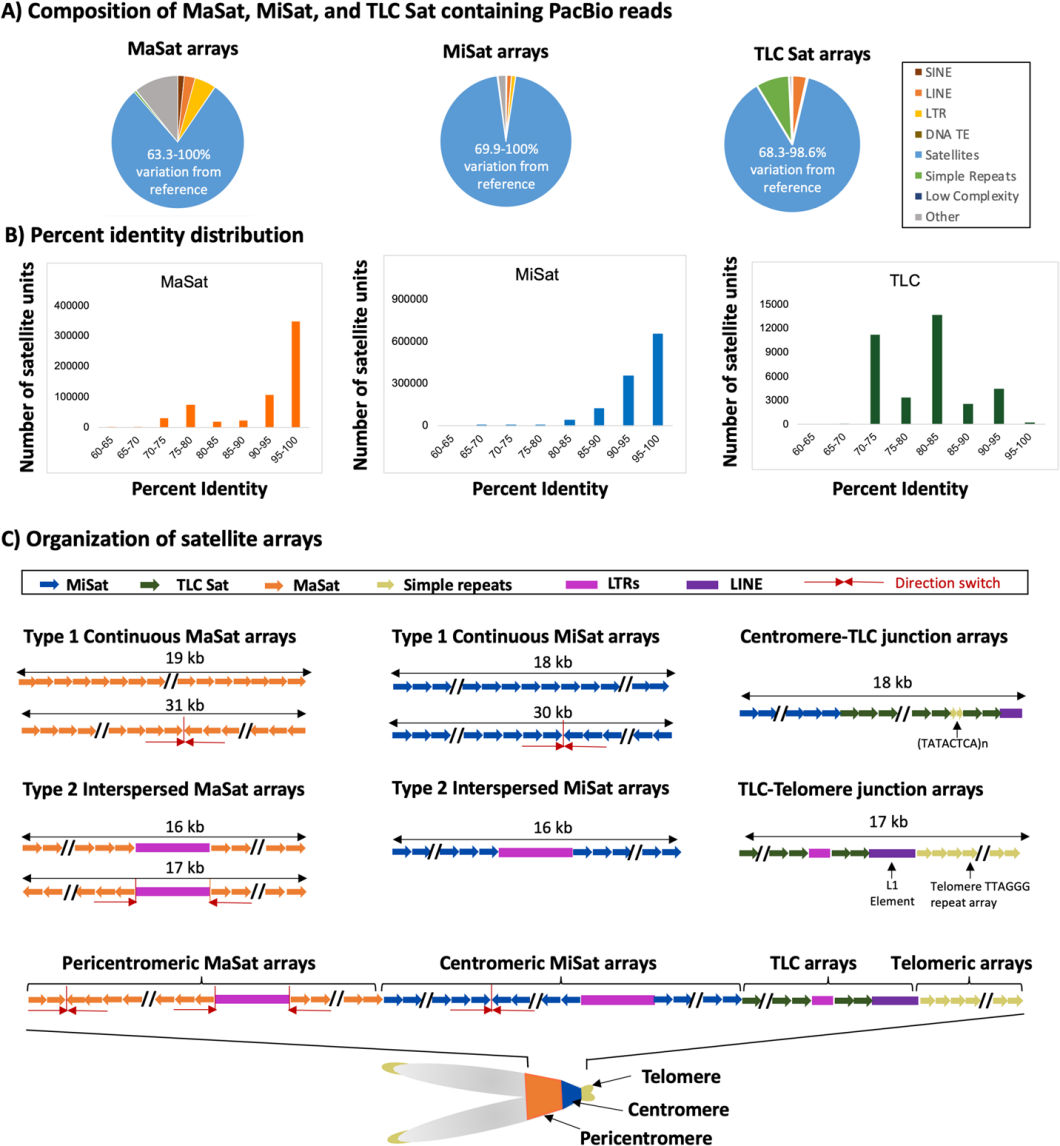
Variations in MiSat and MaSat array composition and organization. **A** Composition of reads containing MaSats (Left), MiSats (Center), and TLC Sats (Right) isolated using BLAST analyses. While MaSats, MiSats, and TLC Sats are the most abundant sequences, non-satellite sequences constitute a significant proportion of these reads. Reference satellite consensus sequences used for BLAST search are as described previously [47, 48, 51]. **B** Percent identity distribution of satellite units for MaSats, MiSats, and TLC Sats within the total pool of satellite units. **C** Organization of representative MaSat and MiSat arrays seen as Type 1 continuous arrays and Type 2 arrays interspersed with non-satellite sequences. Satellite organization at representative Centromere-TLC and TLC-Telomere junctions is shown. Additionally, a schematic for the organization of various satellite and non-satellite sequences from pericentromeric regions to telomeric ends is shown

We analyzed publicly available high coverage LRS data (4.06 million reads, 16.4 kb average read length, ~ 25-fold genome coverage, and 99.8% accuracy) from C56BL/6 J mouse strain generated using PacBio Sequel II System with HiFi sequencing [50]. Using NCBI-BLAST, we identified MaSat, MiSat, and centromere proximal TLC Sat arrays in the LRS data using C56BL/6 reference consensus repeat units as query sequences. The majority of long reads containing MaSat and MiSat ranged from 13 to 19 kb in length (Additional file 1: Fig S1A)). Surprisingly, for each satellite type, individual monomer units within the total satellite pool isolated using BLAST from LRS reads demonstrated sequence divergence up to 63.3%, 69.9%, and 68.3% for MaSats, MiSats, and TLC Sats, respectively (Fig. 1A). Interestingly, the majority of MaSats and MiSats exhibited 90–100% sequence identity, while only a small fraction showed 70–90%. Conversely, for TLC Sats, most exhibited 70–90% sequence identity, with only a small fraction displaying 90–100% (Fig. 1B).

### Mouse centromeric and pericentric regions are organized as continuous and interspersed satellite arrays

Analysis of the LRS reads using RepeatMasker revealed that MaSat and MiSat arrays also contain a significant amount (11.26% in MaSat reads and 4.56% in MiSat reads) of non-satellite sequences (Fig. 1A). These non-satellite sequences include repeats, such as transposable elements, simple repeats, and other unknown sequences. Next, we analyzed the arrangement of satellite and non-satellite sequences on arrays. Satellite containing long reads displayed two distinct organizations: Type 1 continuous arrays (90.05% of MaSat arrays and 98.23% of MiSat arrays) and Type 2 arrays interspersed with non-satellite sequences (10% of MaSat arrays and 1.77% of MiSat arrays) (Fig. 1C, Table 1, Additional file 1: Fig S1B-C). Type 1 continuous MaSat and MiSat arrays included a subset of arrays (0.85% of MaSat arrays and 1.92% of MiSat arrays) where monomers switched direction from forward to reverse or vice-versa (Fig. 1C, Table 1). Type 2 MaSat arrays are mostly interrupted by LTR retrotransposons (31.27%), including those from the IAPEz-int family (ERV2), MTA (ERV3), ERVB4_1B (ERV2), RLTR6 family (ERV1), RLTR10 family (ERV2), and MERVL family (ERV3). Sequences interspersed within Type 2 MiSat arrays predominantly comprised Long Terminal Repeat (LTR) retrotransposons (present in 56.38% of Type 2 MiSat arrays) (Fig. 1C, Table 1) with the IAPEz-int family, part of the intracisternal A-type particle (IAP) class of endogenous retroviruses (ERV2) being the most abundant (Table 1). The IAPEz-int family contains young TEs that have been studied for their roles as functional transcriptional promoters of nearby genes and epigenetic modulators through DNA methylation and H3K9 modifications [52–54]. Another abundant TE interspersed with Type 2 MiSat arrays was the B2 element, which belongs to the Short interspersed nuclear element (SINE) class of non-LTR retrotransposons (Table 1). B2 elements in mice have been shown to be present at boundaries between H3K9me3 and H3K9me2 chromatin domains [55] and provide CCCTC-binding factor (CTCF) binding sites [56, 57].

**Table 1:**
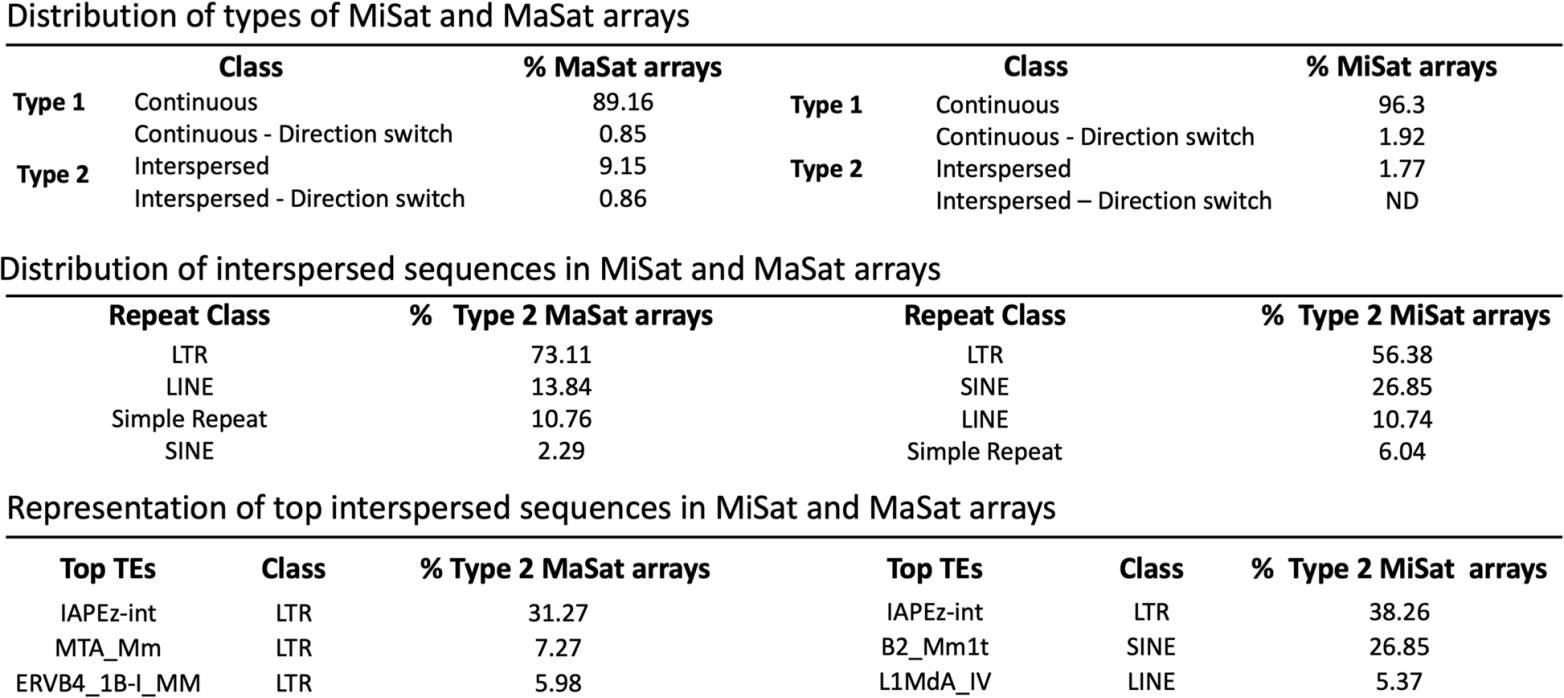
Distribution of types of MiSat (left) and MaSat (right) arrays (top), interspersed sequences in Type 2 MaSat and MiSat arrays (middle) and representation of top interspersed sequences in Type 2 arrays (bottom). These top interspersed sequences are the most frequently occurring non-satellite sequences within the Type 2 MaSat and MiSat arrays

*ND* Not detected

### Mouse centromeric and pericentric satellites exhibit high sequence variations within and across different arrays

**Fig. 2:**
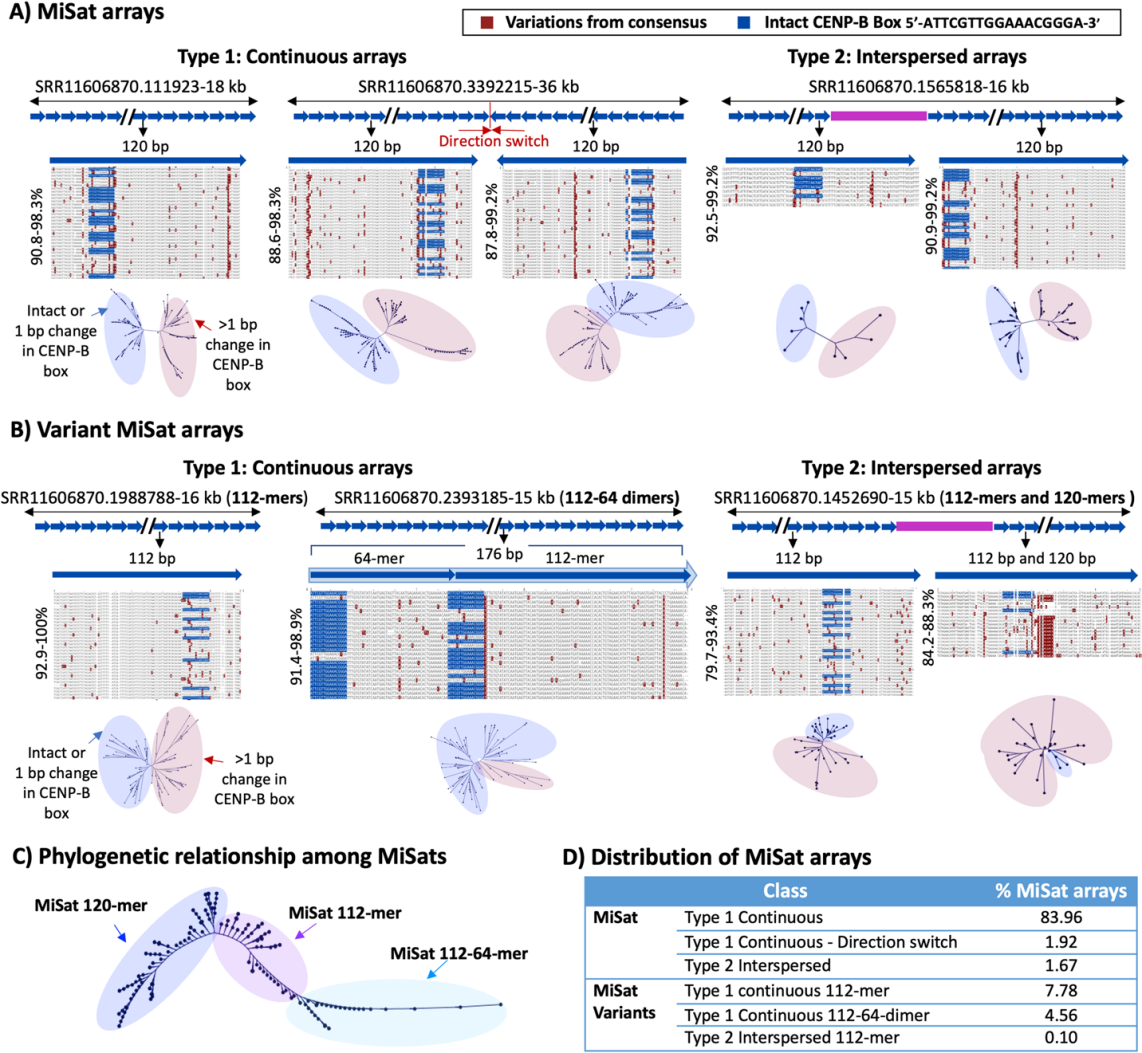
Sequence composition of satellite units within and across centromeric MiSat arrays. Alignment of **A** the MiSat reference sequence with repeat units from representative MiSat arrays and **B** the 112-mer and 112–64-dimer MiSat consensus with repeat units from representative variant MiSat arrays. Reference satellite consensus sequences used for the alignments are as described previously [47, 48, 51]. The length of each array is given, and the* X*-axis is not to the scale. All subunits are arranged in the order they appear, spanning from the beginning to the end of a given array. The alignment of all ordered repeat units with the reference consensus is performed for the entire array. Alignments of repeat units from the beginning of a given array are shown, and alignments for the whole of the arrays are provided in Additional file 2: Fig S2. Neighbor-joining phylogenetic trees illustrating the relationships among all satellite units within a given array are presented below each alignment. **C** The phylogenetic relationship between different classes of MiSats. **D** The distribution of different types of MiSat arrays

To investigate the sequence similarity among repeat units within and across satellite arrays, we compared and aligned satellite monomers isolated from a given LRS read with the *M. musculus* reference MiSat and MaSat satellite units. Strikingly, we found up to 20.3% local nucleotide variation (79.7–99.2% similarity) in MiSat units from the reference on LRS arrays in the C56BL/6 J strain (Fig. 2A–C, Additional file 2: Fig S2). This level of variation is approximately three times higher than the previously reported 5.9% nucleotide variation for MiSats in the C56BL/6 J strain [48]. Similar to human α-satellites, mouse MiSats contain a 17 bp sequence motif called the CENP-B box that binds to CENP-B centromeric protein in a sequence-dependent manner [58–60]. CENP-B is the only centromeric protein that binds to its target satellite sequences in a sequence-dependent manner. Although CENP-B was initially thought to be dispensable for centromere function [61], recent studies have shown its critical role in the maintenance of centromeric memory [62]. We found that most nucleotide changes in MiSats were concentrated at and around the CENP-B box. As a result, an intact CENP-B box was present only in a subset of satellite units in each array (Fig. 2A–B). For Type 2 arrays with interspersed non-satellite sequences, sequence variation was present at either side of the interrupting non-satellite sequence (Fig. 2A). Furthermore, we detected previously known variations in monomer length in MiSats [48].

A subset of MiSat arrays comprises divergent monomers with different monomer lengths: 112-mer (7.78%) and 112–64-dimer (4.56%) (Fig. 2B, D), which were previously reported by Rice (2020) [48]. The density of intact CENP-B Boxes varied greatly between variant 112-mer arrays and 112–64-dimer arrays. Type 1 112-mer arrays contained a few intact CENP-B Boxes, while Type 1 112–64-dimer arrays contained a high number of intact CENP-B Boxes (Fig. 2A, B). Phylogenetic analysis using Neighbor-Joining phylogenetic trees revealed that within each 120-mer, 112-mer, and 112–64-dimeric MiSat array, the repeat units displayed a notable separation into two clades, distinguished by the presence or absence of CENP-B boxes (Fig. 2A and B). The size of these clades correlated with the proportion of repeat units containing or lacking CENP-B boxes. Furthermore, when examining the entire pool of MiSat arrays, the 120-mer, 112-mer, and 112–64-dimeric MiSat classes separated into distinct clades, suggesting a strong evolutionary distinction between these classes (Fig. 2C).

**Fig. 3:**
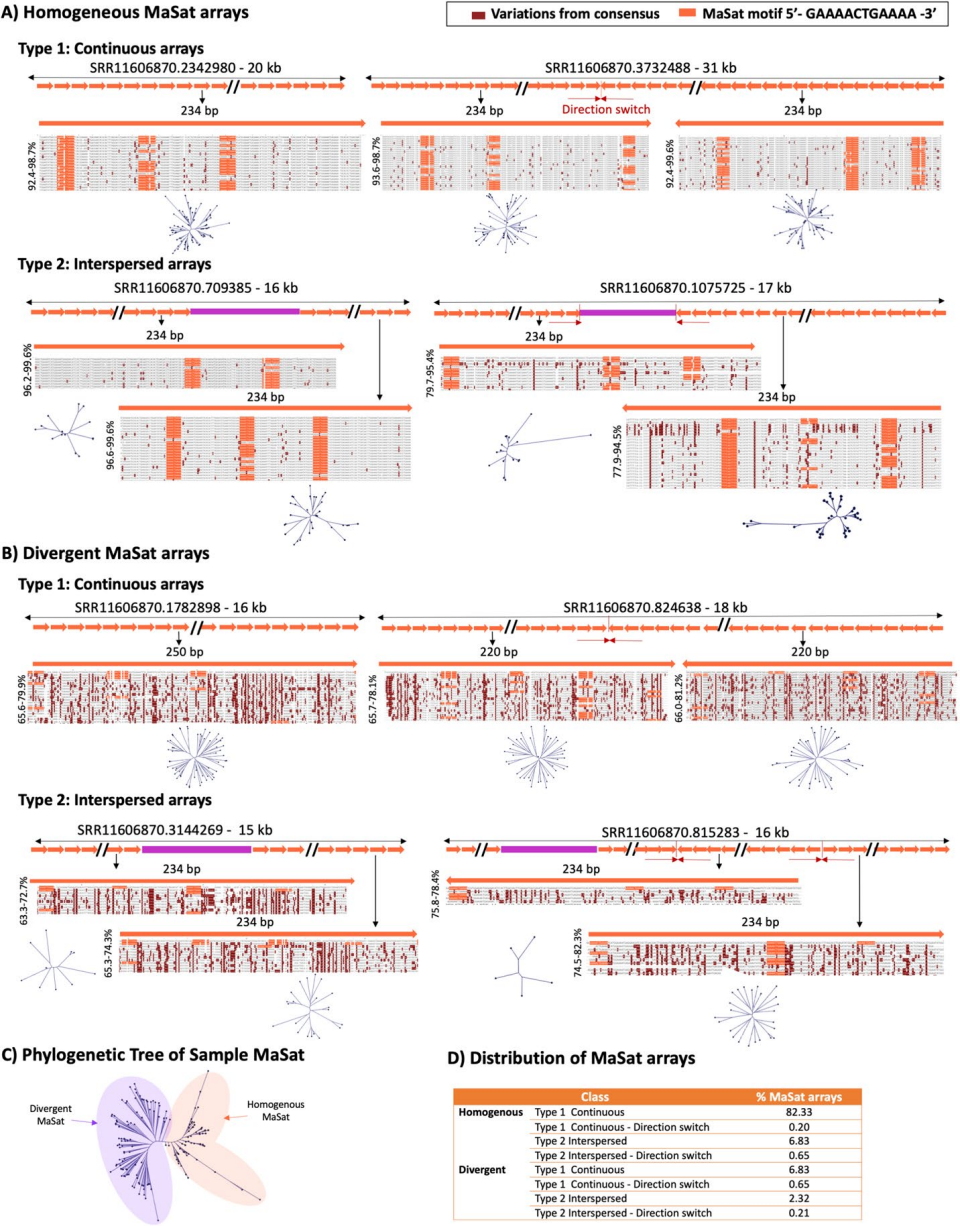
Sequence composition of satellite units within and across pericentromeric MaSat arrays. Alignments of **A** the MaSat consensus with repeat units from four representative MaSat homogeneous arrays, and **B** the MaSat consensus sequences with repeat units from four representative divergent arrays. Reference satellite consensus sequences used for the alignments are as described previously [51]. The length of each array is given, and the *X*-axis is not to the scale. All subunits are arranged in the order they appear, spanning from the beginning to the end of a given array. The alignment of all ordered repeat units with the reference consensus is performed for the entire array. Alignments of repeat units from the beginning of a given array are shown, and alignments for the whole of the arrays are provided in Additional file 3: Fig S3. Neighbor-joining phylogenetic trees illustrating the relationships among all satellite units within a given array are presented below each alignment. **C** The phylogenetic relationship between homogeneous and divergent MaSats. **D** The distribution of different types of MaSat arrays. Phylogenetic trees illustrating the relationships among all satellite units within a given array are provided below each alignment

Subsequently, we observed that the nucleotide variations in arrays containing MaSat were even more pronounced than those in MiSat arrays. MaSats exhibited local nucleotide variations of up to 36.7% from the consensus (63.3–99.6% similarity) on MaSat arrays (Fig. 3A–C, Additional file 3: Fig S3). Based on the level of sequence variations, we categorized MaSats into two types: homogeneous, constituting approximately 90% of MaSats, and divergent, constituting around 10% of MaSats (Fig. 3A–D). Homogeneous Type 1 and Type 2 MaSat arrays contained monomers with up to 22.1% sequence variation from the consensus (77.9–98.7% similarity), within a single array including at MaSat motif 5′- GAAAACTGAAAA -3′ (Fig. 3A, C). Conversely, divergent Type 1 and Type 2 major satellite arrays contained diverged monomers that exhibited up to 36.7% nucleotide variation from the consensus (65.3–79.9% similarity) (Fig. 3B, C).

Nucleotide variations in divergent MaSat arrays included several insertions and deletions, leading to variations in major satellite monomer lengths such as 220-mers and 250-mers (Fig. 3A, B). Phylogenetic analysis using Neighbor-Joining phylogenetic trees revealed that within each Type 1 and Type 2 MaSat array, the repeat units separated into multiple small clades without a discernable pattern (Fig. 3A, B). However, when examining the entire pool of MaSat arrays, homogeneous and divergent MaSats separated into two clades suggesting a significant evolutionary separation between these classes (Fig. 3C).

**Fig. 4:**
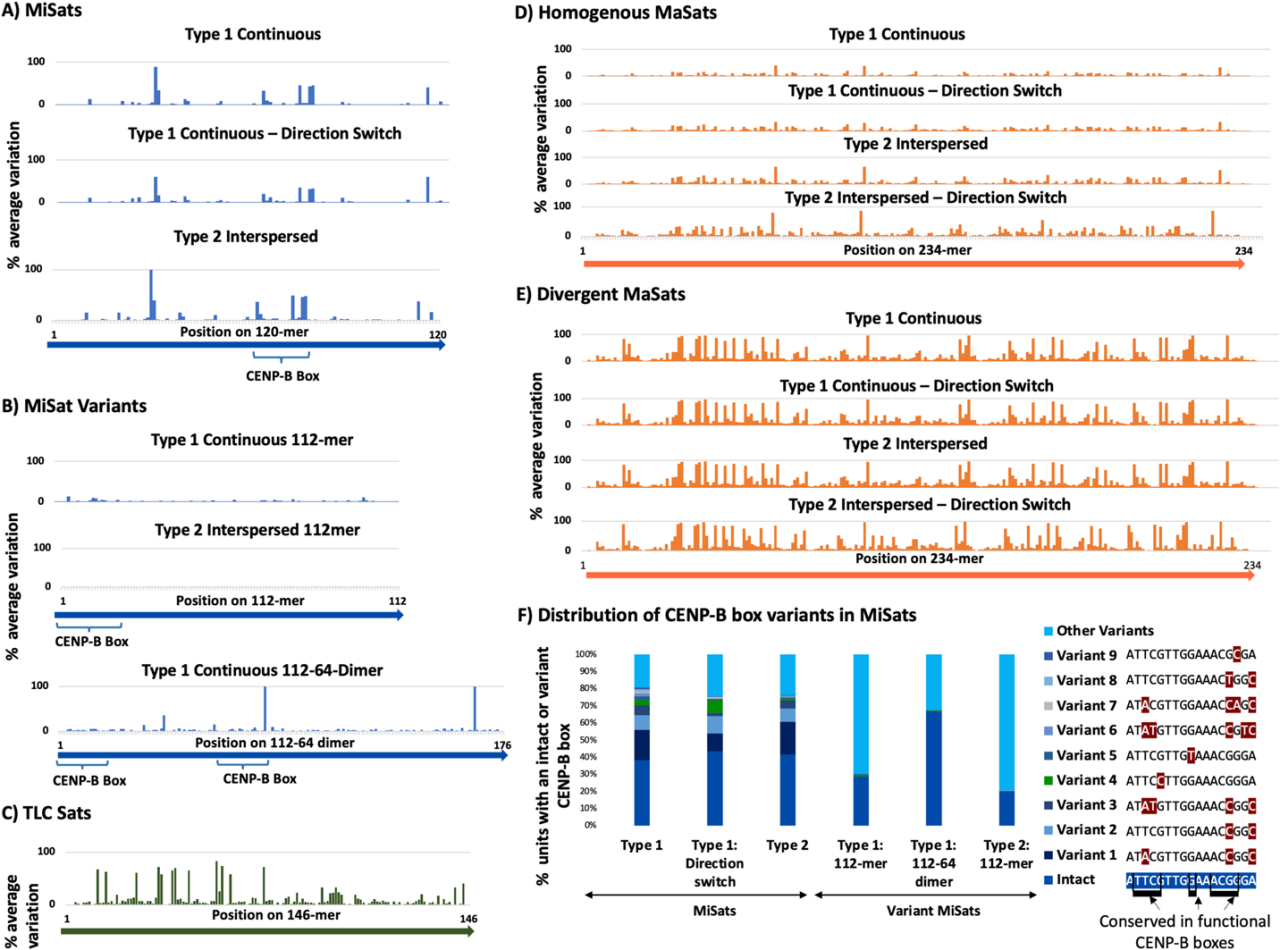
Quantification of single nucleotide polymorphism in satellite units within arrays. **A** Percent average variation at each position of the satellite unit across **A** MiSat Type 1 and Type 2 arrays **B** MiSat variant Type 1 and Type 2 arrays **C** TLC Sat arrays **D** homogeneous MaSat arrays, and **E** divergent MaSat arrays. **F** The distribution of intact and variant CENP-B boxes in different types of MiSat arrays. Bases that have previously been shown to be conserved in functional CENP-B boxes are indicated [59, 64–66]

Next, we analyzed nucleotide variations at each position within the satellite unit for all MiSat, TLC Sat, and MaSat classes (Fig. 4A–E). TLC Sat and MaSat units exhibited high nucleotide variation throughout their respective satellite units (Fig. 4C–E). In contrast, within MiSats, certain positions, particularly those within and around CENP-B boxes, displayed high variability, while the rest of the satellite unit demonstrated high homogeneity (Fig. 4A–B). Specifically, within CENP-B boxes, positions 15–17, known to be conserved in functional CENP-B boxes [62-65] [63], exhibited high sequence variations from the consensus. Consequently, only a subset of satellite units within each class of MiSat arrays contained intact CENP-B boxes, while the remaining units harbored CENP-B box variants (Fig. 4F). Interestingly, among MiSat variants, the 112–64-dimeric MiSat variants exhibited a high percentage (67%) of intact CENP-B boxes, whereas the 112-mer MiSat variants displayed a low percentage (20%) of intact CENP-B boxes (Fig. 4F).

### Abundant 120-mer Misat arrays are preferred as functional centromeres

**Fig. 5:**
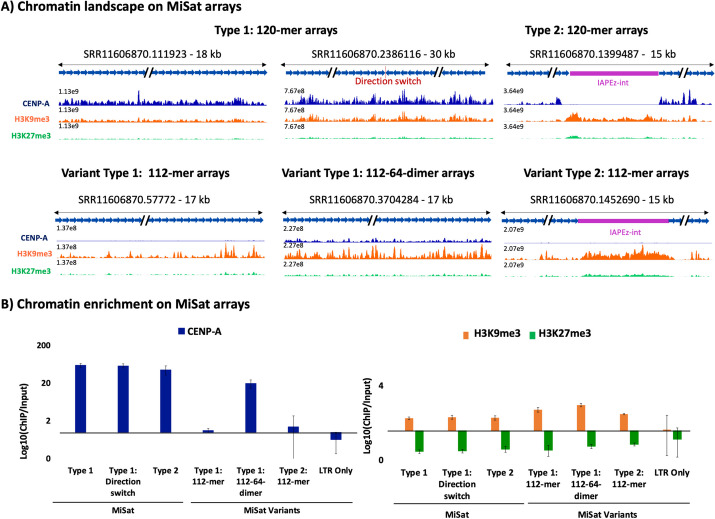
Occupancy of chromatin marks on MiSat arrays. **A** CENP-A, H3K9me3, and H3K27me3 chromatin profiles on representative abundant 120-mer MiSat arrays, and variant 112-mer and 112–64-dimeric MiSat arrays. The *Y*-axis range is set to a constant value for a given array for CENP-A, H3K9me3, and H3K27me3 tracks. The length of each array is given, and the *X*-axis is not to the scale. **B** CENP-A, H3K9me3, and H3K27me3 enrichment calculated by normalizing the ChIP enrichment with the abundance of the respective MiSat array in the ChIP input. The enrichment value was averaged over three or more arrays for each type

To determine if MiSat array types differ in chromatin assembled at mouse centromeric regions, we performed ChIP-seq for CENP-A, H3K9me3, and H3K27me3 in the C56BL/6 J strain. We mapped the sequencing data to representative MiSat array types (Fig. 5A) and calculated enrichment on each array type by normalizing ChIP enrichment with the abundance of the respective array in the ChIP input (Fig. 5B). Among all MiSat types, we observed the highest CENP-A enrichment (up to 60-fold) on abundant 120-mer Type 1 and Type 2 arrays. Within Type 2 interspersed MiSat arrays, CENP-A was enriched at MiSats but not at non-satellite regions, suggesting that TEs interrupting MiSat arrays are not part of functional kinetochores. However, while the IAPEz-int elements interrupting Type 2 MiSat were not enriched in CENP-A, they were significantly enriched in H3K9me3, suggesting that they are repressed at centromeres (Fig. 5A–B). We found that 112-mer MiSat variants showed low (~ 1.5-fold) CENP-A enrichment, while 112–64-dimeric MiSat variants showed moderate (~ 20-fold) CENP-A enrichment (Fig. 5B). Overall, abundant 120-mer Type1 continuous and Type 2 interspersed MiSat arrays are preferred as functional centromeres, as they exhibit high enrichment of the CENP-A, a chromatin mark that targets chromosomal loci for functional centromere formation. Furthermore, a significant enrichment of H3K9me3 on TE elements at centromeric regions indicates their heterochromatic and silenced nature.

### Homogeneous MaSat arrays exhibit increased constitutive heterochromatin at pericentric regions

**Fig. 6:**
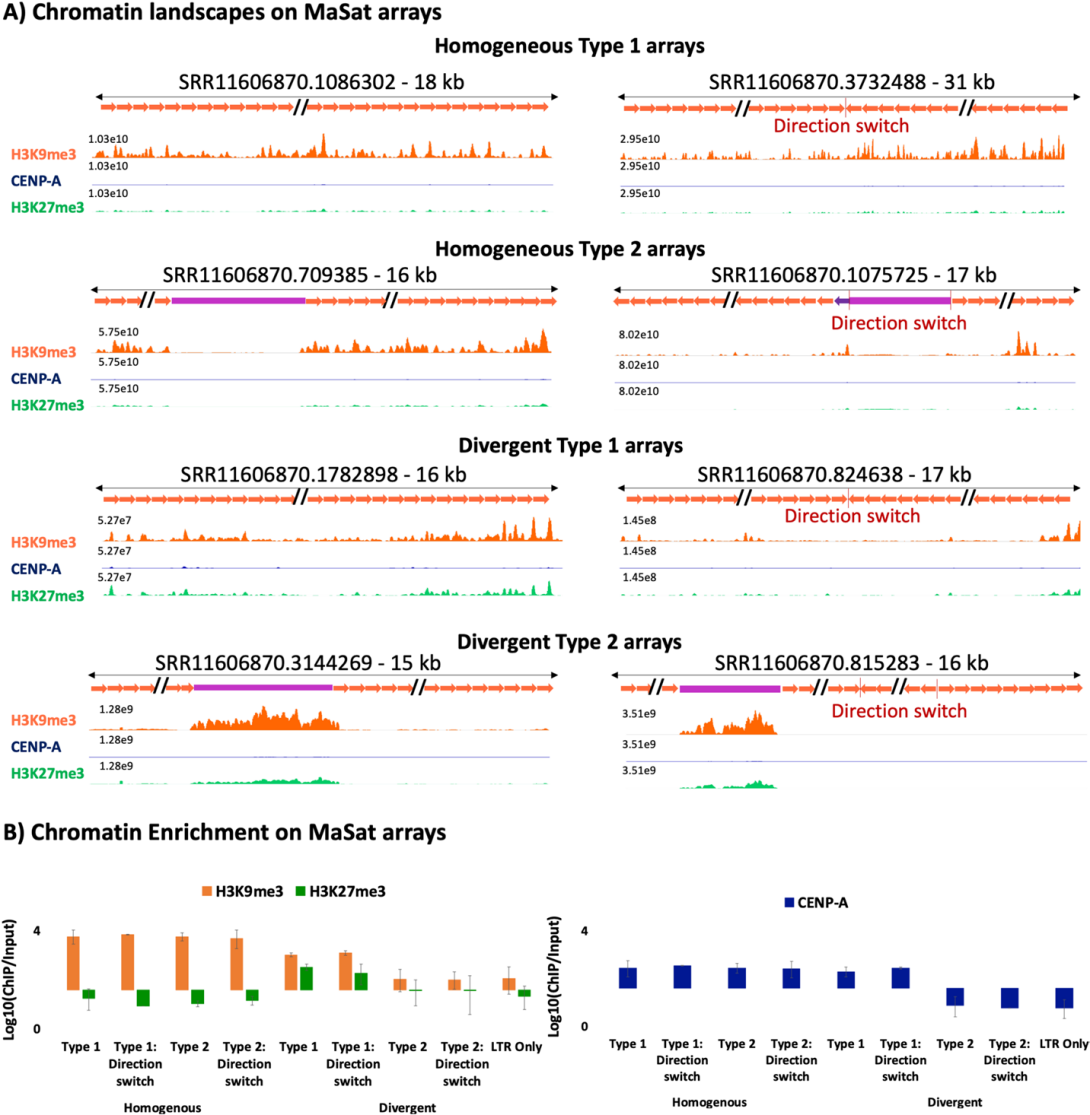
Occupancy of chromatin marks on pericentric MaSat arrays. **A** CENP-A, H3K9me3, and H3K27me3 chromatin profiles on representative homogeneous and divergent MaSat arrays. The Y-axis range is set to a constant value for a given array for CENP-A, H3K9me3, and H3K27me3 tracks. The length of each array is given, and the *X*-axis is not to the scale. **B** CENP-A, H3K9me3, and H3K27me3 enrichment calculated by normalizing the ChIP enrichment with the abundance of the respective MaSat array in the ChIP input. The enrichment value was averaged over three or more arrays for each type

To assess the enrichment of H3K9me3 chromatin in mouse pericentric regions, we aligned the ChIP-Seq data to representative MaSat arrays for both Type 1 and Type 2 arrays (Fig. 6A). In contrast to MiSats, which exhibited high CENP-A enrichment (up to ~ 60 fold), we observed the maximum enrichment of up to ~ 3.5-fold for H3K9me3 on MaSat arrays (Fig. 6A and B). Notably, in most Type 2 MaSat arrays, the interrupting LTR transposon showed a low level (~ 1.5-fold) of H3K9me3 enrichment. However, the H3K9me3 enrichment on flanking MaSats was significantly higher than on the interrupting LTR transposon (Fig. 6A and B).

Furthermore, we found that the H3K9me3 enrichment on divergent MaSat arrays was even lower (~ twofold) compared to homogeneous MaSat arrays. Interestingly, divergent MaSat arrays displayed slightly higher amounts of H3K27me3 facultative heterochromatin compared to their homogeneous counterparts (Fig. 6A and B). In these divergent interspersed arrays, the H3K9me3 enrichment at the interrupting LTR transposon was much higher than the H3K9me3 enrichment at MaSat (Fig. 6A, B). Collectively, these results suggest a strong correlation between sequence homogeneity within MaSat arrays and the presence of constitutive heterochromatin. As sequence homogeneity decreases, MaSat arrays begin to accumulate facultative heterochromatin.

### Local spreading of CENP-A nucleosomes at centromere junctions on both pericentric and telomeric sides

**Fig. 7:**
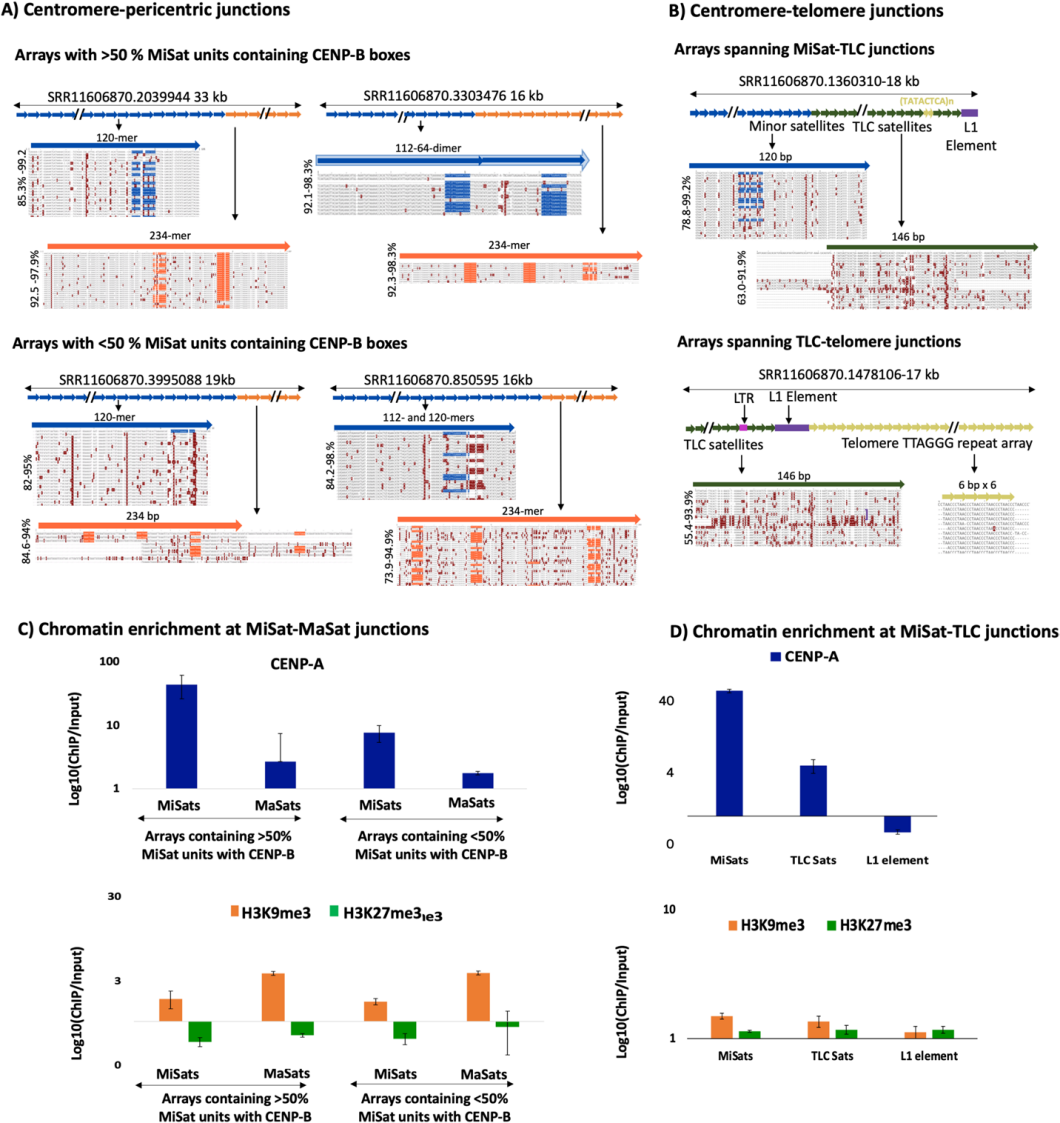
Satellite and chromatin organization at centromere junctions with pericentric and telomeric sides. Alignments of **A** MiSat and MaSat reference consensus sequences with repeat units from representative MiSat-MaSat junction containing arrays. **B** TLC Sat, MiSat, and telomeric repeat consensus with repeat units from representative Cen-Tel junction arrays. Reference satellite consensus sequences used for the alignments are as described previously [47, 48, 51]. The length of each array is given, and the *X*-axis is not to the scale. All subunits are arranged in the order they appear, spanning from the beginning to the end of a given array. The alignment of all ordered repeat units with the reference consensus is performed for the entire array. Alignments of repeat units from the beginning of a given array are shown. **C** CENP-A, H3K9me3, and H3K27me3 chromatin enrichment on MiSat-MaSat and MiSat-TLC Sat junctions calculated by normalizing the ChIP enrichment with the abundance in the ChIP input of the respective MiSat, MaSat or TLC Sat containing part of the junction array. The normalized enrichment value was averaged over three or more arrays for each type

To identify the class of MaSat and MiSat arrays present at centromere-pericentric junctions, we isolated LRS reads containing both MiSats and MaSats and analyzed both sequence composition and chromatin organization on these arrays. MiSats at these junctions belonged to both the MiSat and MiSat variant classes, exhibiting sequence identities ranging from 85.3 to 100% (Fig. 7A). Divergent MiSats present at centromere-pericentric junctions exhibited the lowest density of CENP-B boxes observed in MiSat arrays in this study, with arrays containing as little as 0.02% units with an intact CENP-B box (MiSat-MaSat arrays shown in Fig. 7A, bottom panels). Similarly, MaSats present at centromere-pericentric junctions belonged to both homogeneous and divergent classes. Next, we investigated the satellite organization at the junctions of centromeres and telomeres. Arrays spanning telomere and centromere junctions contained four types of sequences (Fig. 7B): TLC satellites, a short stretch of (TATACTCA)_n_ simple repeats, 5′ truncated L1 element, and telomeric repeats (TTAGGG)_n_ [67]. The 5′ truncated LINE-1/L1 is a previously reported highly conserved element of centromere telomere junctions [42]. L1 is part of the Long interspersed nuclear elements (LINE) group of non-LTR retrotransposons that is highly abundant in almost all mammalian genomes [68]. A few TLC arrays contained LTR such as RLTR17B_Mm. We detected TLC arrays spanning centromere-TLC as well as TLC-telomere junctions. On arrays spanning centromere-TLC junctions, TLC Sat units exhibited a high nucleotide variation (63–91.9%) throughout their respective satellite units (Fig. 7B). On arrays spanning TLC Sats and telomeric repeats, while TLC arrays displayed high nucleotide variation (55.4–93.9%) within a given array, telomeric simple repeats were highly homogeneous (Fig. 7B).

Subsequently, we determined CENP-A, H3K9me3, and H3K27me chromatin enrichment on satellites at centromere junctions on both pericentric and telomeric sides. At centromere-pericentric junctions, homogeneous MiSats, containing > 50% units with an intact CENP-B box, displayed substantial CENP-A enrichment (~ 44-fold), while divergent MiSats, containing < 50% units with an intact CENP-B box, exhibited lower yet significant levels (~ 7.5-fold) of CENP-A enrichment (Fig. 7C, Additional file 4: Fig S4). Additionally, MaSats at centromere-pericentromeric junctions demonstrated a notable amount of CENP-A enrichment (~ 2.7-fold), surpassing CENP-A enrichment (~ 1.5-fold) on randomly selected MaSat arrays from various classes, irrespective of their location on pericentric regions (Figs. 6B and 7C). This suggests a localized spreading of CENP-A chromatin from centromeres to centromere-pericenric junctions (Fig. 7C). MiSats present at centromere and telomere junctions exhibited a high level of CENP-A enrichment (~ 56-fold), while flanking TLC satellites showed a lower (up to ~ fivefold) yet significant amount of CENP-A, implying CENP-A spreading from centromeres to TLC satellites as well (Fig. 7C). Moreover, the 5′ truncated L1 elements at TLC-telomere junctions were depleted for CENP-A (Fig. 7C).

MaSats at centromere-pericentric junctions showed ~ 3.7-fold H3K9me3 levels, comparable to ~ 3.5-fold H3K9me3 enrichment on homogeneous arrays containing only MaSats (Fig. 7C). MiSats at centromere-pericentric junctions displayed ~ 1.7-fold H3K9me3 enrichment, suggesting minimal spreading from adjacent MaSat arrays (Fig. 7C). The L1 elements at the centromere-telomere junctions showed very low levels of H3K9me3 and H3K27me3 enrichment (1.12- and 1.17-fold, respectively), indicating a weak or non-heterochromatic nature of these sites (Fig. 7C). Our findings suggest that CENP-A nucleosomes spread from centromeres to pericentric regions and telomeres. CENP-A chromatin and H3K9me3 heterochromatin also occupy MiSats and MaSats spanning centromeric-pericentric junctions.

### CENP-A and H3K9me3 nucleosomes exhibit distinct organizations and conformations

**Fig. 8:**
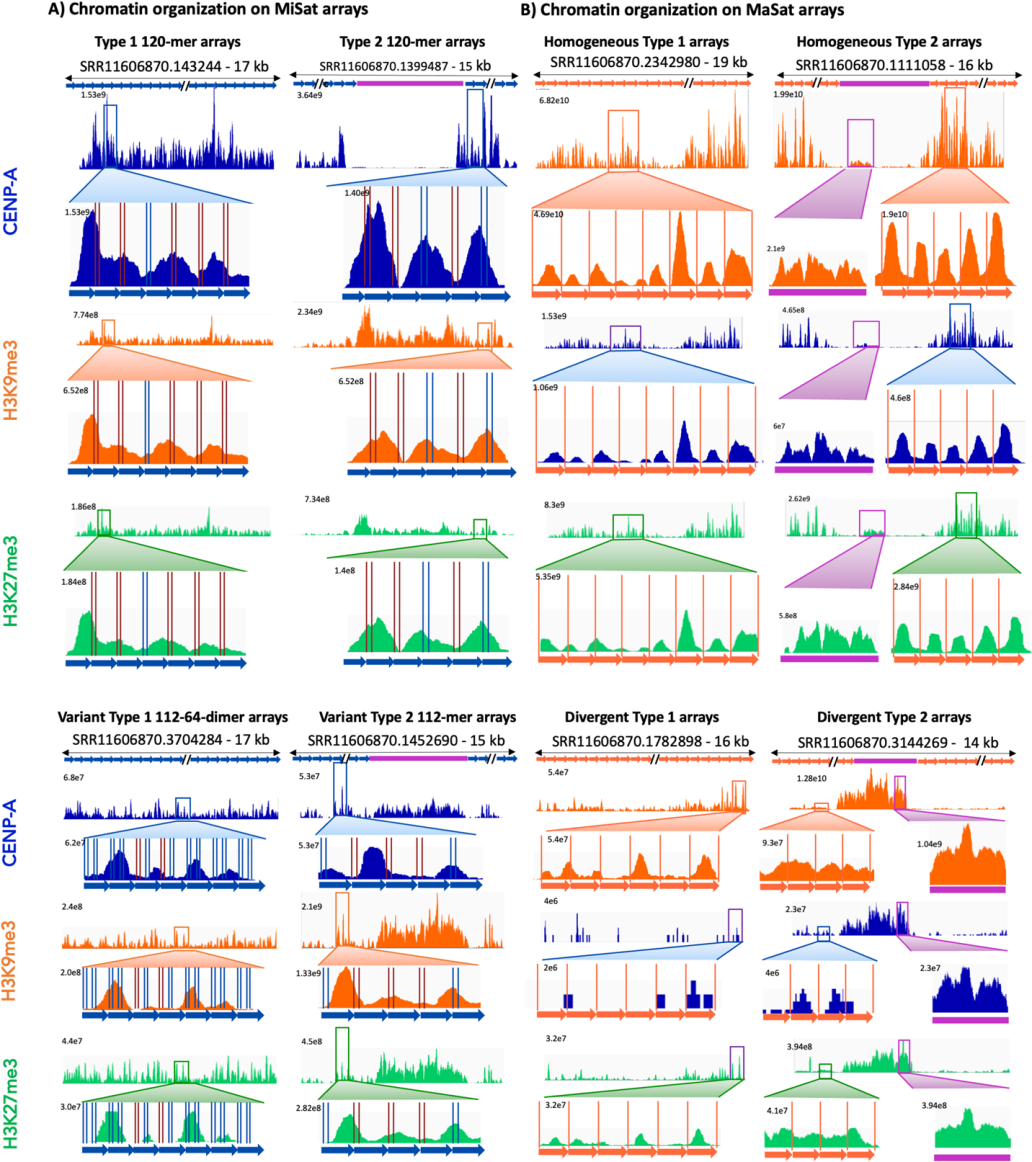
Distinct conformations of CENP-A, H3K9me3, and H3K27me3 containing nucleosomes. **A** CENP-A, H3K9me3, and H3K27me3 ChIP-seq profiles on representative homogeneous and variant Type 1 and Type 2 MiSat arrays. **B** CENP-A, H3K9me3, and H3K27me3 ChIP-seq profiles on representative homogeneous and divergent Type 1 and Type 2 MaSat arrays. CENP-B boxes on MiSat are marked by vertical blue (intact) and red lines (variant). MaSat monomers are separated by orange lines

Next, we analyzed the conformations of CENP-A and H3K9me3 containing nucleosomes on MiSat and MaSat arrays, respectively (Fig. 8A, B). CENP-A chromatin displayed a general lack of nucleosome phasing on MiSat repeat units across all array types (Fig. 8A). CENP-A peaks spanned either a single or multiple tandem MiSats. Although the pattern was not precise, we observed roughly one CENP-A chromatin particle per two MiSat units as the most frequent conformation. These results suggest that mouse CENP-A nucleosomes are tightly associated with other centromeric proteins to form larger complexes similar to those observed on human centromeric α-satellite arrays [22, 23]. Furthermore, CENP-A nucleosome conformations lacked any discernible arrangement relative to the CENP-B box and the variation in CENP-A conformation was not predictable based on the presence of an intact CENP-B box or its variants (Fig. 8A). In contrast, H3K9me3 chromatin on homogeneous and abundant MaSats displayed a relatively uniform conformation with a single peak occupying 234 bp MaSat monomer resulting in a strong nucleosome phasing (Fig. 8B). Interestingly, although CENP-A and H3K27me3 nucleosomes were enriched only slightly on MaSats, they displayed a well-phased conformation, suggesting that homogenous major satellites exhibit the inherent property of phasing all types of nucleosomes (Fig. 8B). Phasing observed in H3K9me3 nucleosomes on MaSat was absent on non-satellite sequences interspersed with MaSat. Divergent MaSat arrays exhibited less H3K9me3 phasing as compared to homogenous MaSat arrays (Fig. 8B). Overall, these results reveal that CENP-A and H3K9me3 nucleosomes have distinct conformations suggesting distinct mechanisms of chromatin assembly at centromeric and pericentromeric regions.

## Discussion

Previous studies have suggested that mouse centromeric MiSats and pericentromeric MaSats of autosomes and the X chromosome are highly homogeneous, with little variation within and across arrays [42, 47]. More recently, Rice (2020) reported an average divergence of 5.9% was reported between minor satellite units at both local and global scales [48]. However, employing 25X high coverage and high-accuracy PacBio data, we observed much higher divergence among centromeric, pericentric, and TLC satellite units at both global and local scales. The previous study employed a robust but relatively narrower approach, focusing on isolating minor satellites by screening for CENP-B boxes and considering only a few polymorphic sites with the CENP-B box [48]. This method excludes divergent minor satellites, which our investigation revealed contain nucleotide changes at multiple sites within the CENP-B boxes. In contrast, our approach to isolating satellite units is more extensive, as it does not depend on screening for CENP-B boxes but considers sequence homology across the entire satellite repeat unit sequence. Consequently, we extracted a comprehensive and broader pool of satellites, demonstrating a much higher range of divergence at both the global and local scales than previously reported. Specifically, we found up to 30%, 36.3%, and 31.6% global variations among the total pool of monomeric repeat units for MiSat, MaSat, and TLC satellites, respectively. At the local level on satellite arrays, we found specific patterns of variations, where some arrays exhibited high homogeneity with up to 92–99.6% sequence identity among repeat units, while others showed high divergence with sequence identity among repeat units as low as 79.7%. For MiSats, sequence variations were notably concentrated at specific positions, including those within the CENP-B box sequence. Notably, although 112–64-mers exhibit organizational differences compared to 120-mers, the divergence between different dimeric units within an array is relatively low, reaching up to 9.6%. This observation parallels the well-known characteristics of human α-satellite higher-order repeats, where adjacent units display divergence while higher-order repeat units maintain higher homology [11, 69]. Conversely, MaSat and TLC Sat units displayed sequence variations distributed throughout their entire length. Nucleotide variations found at the CENP-B box within MiSat arrays included changes that can disrupt the ability of CENP-B to bind the CENP-B box [59]. The presence of numerous CENP-B box variants at mouse centromeres suggests that variant CENP-B boxes might be linked to the differential binding of centromeric proteins on distinct centromeric satellite arrays. We observed that 112-mer MiSat variants with a low density of intact CENP-B boxes exhibited much lower CENP-A enrichment (up to ~ 1.5-fold) compared to the CENP-A enrichment on MiSats (up to ~ 60-fold) containing a high density of intact CENP-B boxes. This supports previous findings that the degeneration of CENP-B boxes in centromeric satellites is associated with a loss in CENP-A binding [22, 24, 48, 69]. While CENP-B was initially considered not essential for centromere function due to the viability of CENP-B knockout mice and the absence of the CENP-B box on neocentromeres and Y centromeres [61, 70], recent studies have shown that lower CENP-B levels are associated with higher missegregation rates and lower fertility, suggesting that CENP-B plays an important role in centromere function and maintenance [62, 71, 72]. Furthermore, the CENP-B box density is correlated with the binding of CENP-A, CENP-B, and CENP-C at human centromeric chromatin [24].

In addition to high sequence variations among satellites within and across arrays, we also identified the presence of TEs at mouse centromeres and pericentromeric regions. This finding is consistent with a previous study that identified TEs, specifically LINE and IAP elements, associated with MiSat [41]. TEs have also been previously identified in centromeric and pericentromeric regions of plants and some eukaryotes, including in humans and *Drosophila* [31, 73–75]. In humans, TEs are predominantly found in the pericentric region [9, 30]. In contrast, in *Drosophila*, islands of retroelements have been found at the functional regions of centromeres that bind CENP-A [31]. The role of TEs at centromeres is not well understood. Some studies have proposed that TEs are drivers of centromere evolution [73, 74]. The formation of new satellites from TE insertions at centromeres offers a potential explanation for the observed rapid evolution of centromeric sequences between species [73–75]. Centromeres have also been proposed to be genomic “safe” insertion zones for TEs, as surrounding repeats can act as a buffer [73, 74]. It has also been speculated that centromeric TEs are transcribed to non-coding RNAs that facilitate CENP-A deposition [74]. While we have identified long stretches of TEs interrupting mouse centromeres similar to *Drosophila*, these TEs do not bind CENP-A themselves. Instead, TEs at mouse centromeres are bound by a low level of repressive constitutive H3K9me3 heterochromatin, suggesting that they are kept in a somewhat silent state to avoid abnormally high transposon activity. Future functional studies will help understand the role of TEs at mouse centromeres. We also observed a low percentage (1.7% and 1.9% of MiSats and MaSats, respectively) of directional switches among arrays suggesting the occurrence of structural rearrangements such as inversion events at both centromeric and pericentric regions.

Previous studies in human centromeres have demonstrated a direct correlation between sequence homogeneity of satellite arrays and high CENP-A enrichment [22, 24, 69]. Our findings indicate a similar pattern for CENP-A enrichment on centromeric and H3K9me3 enrichment on pericentric satellites in mouse. Importantly, while MiSats exhibit high enrichment for CENP-A nucleosomes (up to ~ 60-fold), MaSats show only moderate enrichment for H3K9me3 heterochromatin (up to ~ 3.5-fold). These observations suggest that centromeric satellites are present in limited numbers and are thus utilized more efficiently to ensure sufficient CENP-A binding required for functional centromeres. In contrast, MaSats are highly abundant, constituting up to 10% of the mouse genome [43, 44]. Therefore, only a subset of cells in a population utilizes a given MaSat array.

We found that at centromere-pericentric junctions, MaSats contain a significant CENP-A enrichment (~ 2.7-fold), which suggests a local spreading of CENP-A chromatin to pericentric regions. Similarly, TLC Sats at centromere-telomeric junctions exhibited a significant CENP-A enrichment (~ fivefold). Centromere flanking regions are hotspots for neocentromere formation [76, 77]. Our observation of CENP-A enrichment in regions adjacent to the centromere suggests that the ability of CENP-A to spread locally makes centromere-flanking locations conducive to neocentromere formation when a native centromere is inactivated or deleted. Furthermore, while CENP-A chromatin on MiSats (120 bp monomeric unit) and H3K9me3 heterochromatin on divergent MaSats (234 bp monomeric unit) and TEs lack nucleosome phasing, homogeneous MaSats (234 bp monomeric unit) exhibit striking phasing of all three types of nucleosomes (CENP-A, H3K9me3, and H3K27me3). These findings suggest that nucleosome phasing is an inherent property of homogeneous MaSats and that specific DNA sequences and the monomeric unit length may contribute to nucleosome phasing.

Our findings of a surprising level of diversity in sequence and chromatin organization of mouse centromeric and pericentric satellites within and across arrays indicate a potential conserved pattern of centromeric satellite variations between mice and humans. Although the extent of variations found in human centromere and pericentric regions is higher compared to mouse [9], our findings raise the possibility that mouse genomes may also contain chromosome-specific centromeric satellite arrays. Future studies using cytological analysis techniques will provide insight into the presence of chromosome-specific arrays at mouse centromeres. Additionally, our study highlights the variation in centromeric chromatin structure, even within a single MiSat array as previously seen in humans [24]. The differences in CENP-A organization between adjacent satellite units suggest that small sequence variations might affect the binding of CENP-A. Thus, CENP-A organization and binding in mouse may have a sequence-dependent component. Overall, our findings on the sequence and organization of mouse centromeric satellite and chromatin shed light on the dynamic yet conserved pattern of satellite sequences and organization and provide a basis for future studies on the functional implications of centromeric satellite diversity in mammals.

## Conclusions

Our study reveals extensive variations in DNA sequence and organization within mouse centromeric and pericentric satellite arrays, surpassing previous observations. We find transposable elements are interspersed within centromeric and pericentric satellite arrays. However, those within minor satellite arrays are not part of functional centromeres as they lack significant CENP-A enrichment. Furthermore, we found that while CENP-A chromatin assembled on centromeric minor satellites exhibits poor phasing, H3K9me3 chromatin assembled on the homogenous class of pericentric major satellite arrays is highly phased.

## Methods

### Animals and tissue homogenization

The C57BL/6 strain was purchased from the Jackson Laboratory and maintained following the institutional animal care and use committee guidelines. Liver tissues from euthanized C57BL/6 were snap-frozen in liquid nitrogen and ground to powder using a mortar and pestle. The powder was resuspended in 1 × PBS containing Roche protease inhibitor cocktail (Millipore Sigma Cat# 11,836,170,001) and dounced with a 15-ml glass douncer using 50 strokes on ice. Glass dounced homogenate was further homogenized using the Tekmar homogenizer on ice. The resulting suspension was passed through a 50-micron nylon filter, the flow through was pelleted at 1800 rpm at 4 °C, and the pellet was washed with 1X PBS. The pellet containing homogenized cells was resuspended in 1X PBS.

### Chromatin Immunoprecipitation (ChIP)

Native ChIP was performed using the protocol described previously [23] with a few modifications on homogenized liver cells. Homogenized cells were resuspended in MNase dilution buffer (20 mM Tris–HCl pH8.1, 200 mM NaCl, 2 mM EDTA, 1% Triton X-100, 0.05% SDS, 3 mM CaCl2, 1X cOmplete Protease Inhibitor Cocktail tablets from Roche #11,697,498,001) and digested with MNase (2.5 U per million cells) at 37 °C for 5 min. MNase-digested nuclei were passed through a 26-gauge needle five times and centrifuged at 500 g for 5 min at 4 °C. The supernatant was saved as the 200-mM salt fraction. The pellet was resuspended in MNase digestion buffer containing 350 mM NaCl and incubated at 4 °C on a shaker for 2 h, followed by centrifugation at 10,000 rpm for 5 min at 4 °C. The supernatant was combined with the 200 mM salt fraction and was aliquoted for multiple ChIP assays, each consisting of 10 million cells. For ChIP, antibodies (4 µl) against specific marks: anti-CENP-A (Cell Signaling Technologies, Cat # C51A7), H3K9me3 (Abcam, Cat # ab8898), H3K27me3 (Cell Signaling Technologies, Cat # C36B11), and IgG (Abcam, Cat # ab46540) were added to the soluble supernatant and incubated overnight at 4 °C. Subsequently, 50 µl of Protein A magnetic beads (New England Biolabs # S1425S) were added to each reaction and incubated for 1 h at 4 °C. The chromatin-antibody-bead complexes were washed five times with MNase digestion buffer containing 200 mM NaCl. After washing, the complexes were resuspended in 700 µl MNase digestion buffer. Next, DNA was extracted from isolated chromatin complexes containing solution by adding 4 µl RNase, followed by a 20-min incubation at 37 °C. Subsequently, 3.5 µl of 10% SDS and 7.5 µl Prot K were added, and the mixture was incubated at 55 °C for 30 min. Phenol/chloroform extraction was performed, and 1 µl glycogen and 1 ml ethanol were added for overnight incubation at − 20 °C. Samples were centrifugated at 13,000 rpm for 30 min, a 70% ethanol wash was performed, and the DNA was resuspended in 45 µl 0.1X TE.

### Library preparation and sequencing

Libraries were prepared from ChIP DNA fragments using the KAPA HyperPrep Kit following the KAPA HyperPrep Kit manual. The library was sequenced using the NextSeq 500/550 Mid Output Kit to generate paired-end 75 bp reads for each sample.

### Analysis of chromatin profiling data

The sequencing reads from CENP-A, H3K9me3, and IgG ChIP sequencing were mapped to sample minor and major satellite containing LRS reads using Bowtie2 (bowtie2 –end-to-end –very-sensitive –no-mixed –no-discordant -q –phred33 -I 10 -X 700) [78].The sam files generated by Bowtie2 were converted to bed files using samtools and bedtools. The bedgraphs were generated using a custom script and visualized on the Integrated Genome Viewer (IGV) [79].

### Analysis of long-read HiFi sequencing data

We analyzed long-read HiFi sequencing data generated using the PacBio Sequel II system for C57BL/6 J mouse genome from Hon et al. (2020) [50]. The read length distribution of all reads was calculated using BBMap global aligner from the Joint Genome Institute (readlength.sh bin = 500) [76]. To isolate major and minor satellite arrays, the LRS data was searched against libraries of *Mus musculus* major and minor satellite reference sequences using NCBI BLAST (blastn -query -word_size 6 -evalue 1e-10 -dust no -outfmt 6). The read length distributions of reads with minor and major satellites were calculated using BBMap: readlength.sh bin = 500. The long reads identified to contain at least one minor or major satellite were then further searched against the RepeatMasker database to characterize TEs and other repeats in the arrays (RepeatMasker -species “Mus musculus” -a). MiSat 112-mer and 112–64-mer variants were identified by searching MiSat long reads against reference sequences [48] using NCBI BLAST (blastn -query -word_size 6 -evalue 1e-50 -dust no -outfmt 6). MaSat arrays containing greater than 10 repeats with less than 75% sequence similarity to *Mus musculus* major satellite reference sequence were classified as Divergent MaSat arrays. CENP-B box sequences from all minor satellites were extracted and clustered using CD-HIT (cd-hit-est -c 1.0 -n 10) to analyze CENP-B box variants [77, 80]. Sample satellite containing arrays (36 minor, 6 centromere-telomere junctions, and 39 major) identified from the LRS data were selected for further analysis. Minor and major satellite monomers were isolated from sample arrays using TideHunter (TideHunter –max-diverg 0.40) [81]. The satellite monomers from sample arrays were aligned in order using default parameters on DNADynamo (BlueTractor Software Ltd), and *M. musculus* reference satellite sequences were added to the top of the alignments. Any alignment errors were corrected manually on DNADynamo and visualized with multiple alignment viewer Mview [82]. Phylogenetic trees of sample array alignments were constructed using the Neighbor-Joining method with the nucleotide distance measure set to Jukes-Cantor in CLC Sequence Viewer. Phylogenetic trees for all MiSat and MaSat subclasses were constructed with the same parameters using a random sample of 30 sequences from each subclass. To determine the sequence variation at each position of a given satellite, satellite units isolated from BLAST were mapped to reference sequences using Bowtie2. The sam files generated by Bowtie2 were converted to bam files using samtools. The bam files were analyzed using Bam-readcount to determine the variation at each position of the satellite unit.

### Supplementary Information

**Additional file 1:** **Fig S1. **A) Read length distribution in the LRS data analyzed in this study. Detailed organization of sample LRS reads with B) MiSat arrays, and C) MaSat arrays.

**Additional file 2:** **Fig S2. **Full alignments of repeats units from MiSat arrays with the reference consensus sequence. The length of each array is given, and the X-axis is not to the scale. All subunits are arranged in the order they appear, spanning from the beginning to the end of a given array. The alignment of all ordered repeat units with the reference consensus is performed for the entire array.

**Additional file 3:** **Fig S3. **Full alignments of repeat units from MaSat arrays with the reference consensus sequence. The length of each array is given, and the X-axis is not to the scale. All subunits are arranged in the order they appear, spanning from the beginning to the end of a given array. The alignment of all ordered repeat units with the reference consensus is performed for the entire array.

**Additional file 4:** **Fig S4. **CENP-A, H3K9me3, and H3K27me3 ChIP-seq profiles on representative arrays from centromeric-pericentric and centromeric-telomeric junction arrays. The Y-axis range is set to a constant value for a given array for CENP-A, H3K9me3, and H3K27me3 tracks. The length of each array is given, and the X-axis is not to the scale.

**Additional file 5.** Review history.

## Data Availability

Datasets produced in this study are submitted to Sequence Read Archive under BioProject Accession number PRJNA979118 [83]. PacBio dataset analyzed in this study is from [50] is available at Sequence Read Archive under accession SRR11606870 [84].
