# Supplementary material for: DNA satellite and chromatin organization at mouse centromeres and pericentromeres
Source: Genome Biol. 2024 Feb 20;25:52. doi: 10.1186/s13059-024-03184-z (PMC10880262; doi:10.1186/s13059-024-03184-z)
Supplement: Supplementary file 1 — Additional file 1: Fig S1. A) Read length distribution in the LRS data analyzed in this study. Detailed organization of sample LRS reads with B) MiSat arrays, and C) MaSat arrays. [file 13059_2024_3184_MOESM1_ESM.docx]

**Additional file** **1: Fig S1.** A) Read length distribution in the LRS data analyzed in this study. Detailed organization of sample LRS reads with B) MiSat arrays, and C) MaSat arrays.
