# Supplementary material for: DNA satellite and chromatin organization at mouse centromeres and pericentromeres
Source: Genome Biol. 2024 Feb 20;25:52. doi: 10.1186/s13059-024-03184-z (PMC10880262; doi:10.1186/s13059-024-03184-z)
Supplement: Supplementary file 2 — Additional file 2: Fig S2. Full alignments of repeats units from MiSat arrays with the reference consensus sequence. The length of each array is given, and the X-axis is not to the scale. All subunits are arranged in the order they appear, spanning from the beginning to the end of a given array. The alignment of all ordered repeat units with the reference consensus is performed for the entire array. [file 13059_2024_3184_MOESM2_ESM.docx]

**Additional file** **2: Fig S2.** Full alignments of repeats units from MiSat arrays with the reference consensus sequence. The length of each array is given, and the X-axis is not to the scale. All subunits are arranged in the order they appear, spanning from the beginning to the end of a given array. The alignment of all ordered repeat units with the reference consensus is performed for the entire array.
