# Supplementary material for: DNA satellite and chromatin organization at mouse centromeres and pericentromeres
Source: Genome Biol. 2024 Feb 20;25:52. doi: 10.1186/s13059-024-03184-z (PMC10880262; doi:10.1186/s13059-024-03184-z)
Supplement: Supplementary file 3 — Additional file 3: Fig S3. Full alignments of repeat units from MaSat arrays with the reference consensus sequence. The length of each array is given, and the X-axis is not to the scale. All subunits are arranged in the order they appear, spanning from the beginning to the end of a given array. The alignment of all ordered repeat units with the reference consensus is performed for the entire array. [file 13059_2024_3184_MOESM3_ESM.docx]

**** **Additional file** **3: Fig S3.** Full alignments of repeat units from MaSat arrays with the reference consensus sequence. The length of each array is given, and the X-axis is not to the scale. All subunits are arranged in the order they appear, spanning from the beginning to the end of a given array. The alignment of all ordered repeat units with the reference consensus is performed for the entire array.
