# Supplementary material for: DNA satellite and chromatin organization at mouse centromeres and pericentromeres
Source: Genome Biol. 2024 Feb 20;25:52. doi: 10.1186/s13059-024-03184-z (PMC10880262; doi:10.1186/s13059-024-03184-z)
Supplement: Supplementary file 4 — Additional file 4: Fig S4. CENP-A, H3K9me3, and H3K27me3 ChIP-seq profiles on representative arrays from centromeric-pericentric and centromeric-telomeric junction arrays. The Y-axis range is set to a constant value for a given array for CENP-A, H3K9me3, and H3K27me3 tracks. The length of each array is given, and the X-axis is not to the scale. [file 13059_2024_3184_MOESM4_ESM.docx]

**Additional file** **4: Fig S4.** CENP-A, H3K9me3, and H3K27me3 ChIP-seq profiles on representative arrays from centromeric-pericentric and centromeric-telomeric junction arrays. The Y-axis range is set to a constant value for a given array for CENP-A, H3K9me3, and H3K27me3 tracks. The length of each array is given, and the X-axis is not to the scale.
