# Supplementary material for: DNA satellite and chromatin organization at mouse centromeres and pericentromeres
Source: Genome Biol. 2024 Feb 20;25:52. doi: 10.1186/s13059-024-03184-z (PMC10880262; doi:10.1186/s13059-024-03184-z)
Supplement: Supplementary file 5 — Additional file 5. Review history. [file 13059_2024_3184_MOESM5_ESM.docx]

**1^st^ round**

**Reviewer 1**

Summary:

This manuscript investigates the organization of satellite DNA and chromatin at mouse centromeres and pericentromeres using published long-read sequencing data and new ChIP-seq data to investigate CENP-A and H3K9me3 chromatin organization. The organization of MiSats and MaSats was poorly understood due to sequencing and repetitive genomic regions challenges. The authors find variations in both sequence and organization of MiSats and MaSats within and across arrays in the genome and identify transposable elements (TEs) interspersed with satellites. ChIP-seq data shows the enrichment of CENP-A and H3K9me3 at both centromeric and pericentromeric regions and absence of CENP-A at centromeric TEs. The main finding centers around the significant variation observed between repeats and across arrays of minor and major satellites. However, the study would greatly benefit from more quantitative analysis of these variations.

Comments:

1) Figure 1: The schematic for mouse chromosome in figure 7 could be included in figure 1 to help the reader visualize the centromeric and pericentromeric regions discussed throughout the paper.
2) The authors focus their paper on the observation of surprising variation across repeats and within arrays. However, the authors do not define what they mean by "significant variation" and present the data in the form of alignments to a consensus. The reader is expected to infer the abundance of such variation, but it's unclear what % of repeats are variants in general and for specific array types (for instance, in the most abundant type of array which is the continuous type in both MiSat and MaSat). What % of individual repeats contains SNPs? What % of an array contains variant repeats? Data visualization with something like deepTools would more effectively represent the variation.
3) How do variants relate to one another evolutionarily? Neighbor-joining trees could be used for this.

4) Another key statement is that much of the variation falls within CENP-B. But again, this needs to be inferred by alignments, as no quantitative representation of abundance of SNPs at any given position within a repeat is shown. A plot showing frequency of NPS along positions within the consensus of sat repeats could show this more effectively and quantitatively.
5) Rice (2020, preprint) analyzed the length and sequence polymorphism of centromeric repeats found on the X and the autosomes, showed high heterogeneity in monomer sequence at both local and global levels, identified variation in CENP-B boxes, and analyzed patterns of CENP-A enrichment. The array variants shown in figure 2B of this paper were also identified by Rice (2020). One thing this previous study did not discuss was telomeric-centromeric junctions. And this study found more variation than Rice (2020) paper. The authors should discuss their findings in the context of this recent study, even though as far as I can tell it remains a preprint.
6) In the discussion, the authors state that "the extent of variations found in human centromere regions is higher compared to mice". How do the authors draw this conclusion? What kind of comparisons did they do and could the include such comparisons?

7) The authors should use a consistent color scheme for all figures showing TLC satellites and Telomeric satellites. Also, the colors used for the different repeats are in some cases hard to distinguish (e.g. two different shades of darker blue for two different repeats)

8) For the centromere/telomere junctions, where is MiSat and why is it not shown? Is there a gap in the LRS data at the junction? Since the authors discuss the centromere/telomere junction, is unclear where the "centromere" part is supposed to be and what sequences are present at the transition.

9) Figure 2 & 3: As mentioned before, sequence variation should be shown in a more quantitative way. Showing genome browser alignments is only partially informative.
10) The alignment screenshots are positioned below individual repeats from what appear to be specific parts of the array. It's not clear what the arrows connecting the alignments and the repeats mean. Do the authors know that the variants shown below come from, say the left of the array? What is the basis for how are the variants grouped together for each array? This figure is quite confusing. As suggested in a previous comment, a more comprehensive and quantitative representation of the data would be preferred (e.g. deepTools)

11) It it not clear what does the y-axis represent in 2E. It can be explained in figure legend.

12) The authors mention in the text that some of the CENP-B box variants are predicted to not bind CENP-B. Which variants are not functional? What % of CENP-B boxes are predicted to be not functional for each array? This data would be interesting to correlate with abundance of CENP-A in each array to determine if CENP-B and CENP-A occupancies are positively correlated.

13) Figure 4 & supp fig 4: Again, TLC junction should be defined so readers can understand where the CENP-A and MiSat are present relative to the telomere.

14) Typo in Figure 6A 'Cchromatin organization'

15) Figure 7 is not cited anywhere in the text.

16) On Page 12, the authors state "At centromere and telomere junctions, TLC satellites were not enriched for CENP-A or H3K9me3 (Figure 4B, Supplementary Figure 4)." But the supplementary figure 4 shows a zoomed region on TLC with CENP-A and H3K9me3 peaks.

17) Something that I thought was missing in this manuscript is a discussion of the observation that CENP-A and H3K9me3 co-exist on MiSat. This is somewhat surprising based on fiber-IF data suggesting that H3K9me3 is not on the CENP-A region in human cells. Discussion of what we know about the two types of chromatin and how they are arranged in other system (with the caveat being that in some cases genomic data are lacking). Since the authors have data for nucleosome phasing, they can also speculate about why CENP-A and H3K9me3 are observed in the same positions, which could be explained by bulk-chromatin analysis afforded by ChIP-seq (e.g. in some cells CENP-A is present at a given position, in others H3K9me3, which would go against a sequence-specific binding pattern for CENP-A proposed by the authors in the discussion).
18) The authors should discuss phasing of CENP-A in other systems, when known.

19) The authors should include more details about the ChIP-seq methods rather than simply citing other papers.

**Reviewer 2**

The work presented by Packiaraj and Thakur on "DNA satellite and chromatin organisation at house mouse centromeres and pericentromeres" is well structured and presents innovative results that deserve publication, not least because these sequences have been neglected so many times. More recent data emphasize their importance for understanding the biology of chromosomes and the genome.

Recent sequencing technologies bring new opportunities to study these complex and difficult sequences. The results of this work are innovative enough to be published in this important journal:

- Both centromeric minor satellites and pericentromeric major satellites exhibited sequence variations within and between arrays;
- Enrichment of CENP-A and H3K9me3 at both centromeric and pericentromeric regions differs on arrays containing different satellite variants;
- TEs at centromeric regions were not bound to CENP-A, indicating their absence from the functional centromeric domains.
- MaSat arrays contain H3K9me3 nucleosomes in a well-phased configuration, while MiSat arrays contain CENP-A nucleosomes that lack a phased configuration.

All this evidence may in fact reveal the structure needed to form a centromere, or rather a functional centromere.

**Authors’ response**

Reviewer #1

This manuscript investigates the organization of satellite DNA and chromatin at mouse centromeres and pericentromeres using published long-read sequencing data and new ChIP-seq data to investigate CENP-A and H3K9me3 chromatin organization. The organization of MiSats and MaSats was poorly understood due to sequencing and repetitive genomic regions challenges. The authors find variations in both sequence and organization of MiSats and MaSats within and across arrays in the genome and identify transposable elements (TEs) interspersed with satellites. ChIP-seq data shows the enrichment of CENP-A and H3K9me3 at both centromeric and pericentromeric regions and absence of CENP-A at centromeric TEs. The main finding centers around the significant variation observed between repeats and across arrays of minor and major satellites. However, the study would greatly benefit from more quantitative analysis of these variations.

Authors: We thank the reviewer for several excellent suggestions and for bringing critical oversights to our attention. Addressing these points by performing further analyses and providing additional information in the revised manuscript has helped us provide a better narrative of our findings with comprehensive evidence.

Comments:

1) Figure 1: The schematic for mouse chromosome in figure 7 could be included in figure 1 to help the reader visualize the centromeric and pericentromeric regions discussed throughout the paper.

Authors: As per the suggestion, we have now included the schematic illustrating mouse centromeres, pericentromeres, and telomeres in Figure 1 to aid in visualizing these regions discussed throughout the paper.

2) The authors focus their paper on the observation of surprising variation across repeats and within arrays. However, the authors do not define what they mean by "significant variation" and present the data in the form of alignments to a consensus. The reader is expected to infer the abundance of such variation, but it's unclear what % of repeats are variants in general and for specific array types (for instance, in the most abundant type of array which is the continuous type in both MiSat and MaSat). What % of individual repeats contains SNPs? What % of an array contains variant repeats? Data visualization with something like deepTools would more effectively represent the variation.

Authors: We thank the reviewer for pointing out this major oversight. We have now included exact variation for the total pool of each type of satellite (Figure 1B) and for every array presented in Figures 2, 3 & 7. For each satellite type, individual monomer units within the total satellite pool isolated using BLAST from LRS reads exhibited sequence divergence up to 63.3%, 69.9%, and 68.3% for MaSats, MiSats, and TLC Sats, respectively (Figure 1A). Interestingly, the majority of MaSats and MiSats exhibited 90-100% sequence identity, while only a small fraction showed 70-90%. Conversely, for TLC Sats, most exhibited 70-90% sequence identity, with only a small fraction displaying 90-100% (Figure 1B). Furthermore, a new figure (Figure 4) has been incorporated to quantitatively represent sequence polymorphism, illustrating percent changes at each position of the satellite unit for all classes of arrays. Specifically, we analyzed nucleotide variations at each position within the satellite unit for all MiSat, TLC Sat, and MaSat classes (Figure 4). TLC Sat and MaSat units exhibited high nucleotide variation throughout their respective satellite units (Figure 4C-4E). In contrast, within MiSats, certain positions, particularly those within and around CENP-B boxes, displayed high variability, while the rest of the satellite unit demonstrated high homogeneity (Figure 4A-4B). Within CENP-B boxes, positions 15-17, known to be conserved in functional CENP-B boxes, exhibited high sequence variations from the consensus. Therefore, only a subset of satellite units within each class of MiSat arrays contained intact CENP-B boxes, while the remaining units harbored CENP-B box variants (Figure 4F).

3) How do variants relate to one another evolutionarily? Neighbor-joining trees could be used for this.

Authors: We have now included the phylogenetic relationships within and between satellite variant arrays. Phylogenetic analysis using Neighbor-Joining phylogenetic trees revealed that within each 120-mer, 112-mer, and 112-64-dimeric MiSat array, the repeat units displayed a clear separation into two clades, distinguished by the presence or absence of CENP-B boxes (Figure 2A and 2B). The size of these clades correlated with the proportion of repeat units containing or lacking CENP-B boxes. Furthermore, when examining the entire pool of MiSat arrays, the 120-mer, 112-mer, and 112-64-dimeric MiSat classes separated into distinct clades, suggesting a sharp evolutionary distinction between these classes (Figure 2C).

For Major satellites, within each Type 1 and Type 2 MaSat array, the repeat units separated into multiple small clades without a discernable pattern (Figure 3A, 3B). However, when examining the entire pool of MaSat arrays, homogeneous and divergent MaSats separated into two clades, suggesting a significant evolutionary separation between these classes

4) Another key statement is that much of the variation falls within CENP-B. But again, this needs to be inferred by alignments, as no quantitative representation of abundance of SNPs at any given position within a repeat is shown. A plot showing frequency of NPS along positions within the consensus of sat repeats could show this more effectively and quantitatively.

Authors: As pointed in response to Point 2 above, we have now included percent SNPs on each position, including the CENP-B box in Figure 4.

5) Rice (2020, preprint) analyzed the length and sequence polymorphism of centromeric repeats found on the X and the autosomes, showed high heterogeneity in monomer sequence at both local and global levels, identified variation in CENP-B boxes, and analyzed patterns of CENP-A enrichment. The array variants shown in figure 2B of this paper were also identified by Rice (2020). One thing this previous study did not discuss was telomeric-centromeric junctions. And this study found more variation than Rice (2020) paper. The authors should discuss their findings in the context of this recent study, even though as far as I can tell it remains a preprint.

Authors: We have included a detailed discussion on Rice's findings concerning minor satellite units, particularly the coexistence of 64-mers with 112-mers and the report of an average divergence of 5.9% between minor satellite units at both local and global scales (Introduction section, Page 4, first paragraph, Results section, Page 7, last paragraph, Discussion section, Page 19, first paragraph). Rice 2020 employed a robust but slightly narrower approach, isolating minor satellites by screening for CENP- B boxes and considering only a few polymorphic sites with the CENP-B box [48]. This method excludes divergent minor satellites, which our investigation revealed contain nucleotide changes at multiple sites within the CENP-B boxes. In contrast, our approach to isolating satellite units is more extensive, as it does not depend on screening for CENP-B boxes but considers sequence homology across the entire satellite repeat unit sequence. Consequently, we extracted a comprehensive and broader pool of satellites, demonstrating a much higher range of divergence at both the global and local scales than reported by Rice 2020. Specifically, we found up to 30%, 36.3%, and 31.6% global variations among the total pool of monomeric units for MiSat, MaSat, and TLC satellites, respectively (Now shown in Figure 1A and 1B). At the local level on satellite arrays, we found specific patterns of variations, where some arrays exhibited high homogeneity with up to 92-99.6% sequence identity among repeat units, while others showed high divergence with sequence identity among repeat units as low as 79.7% (now depicted in Figure 2 and Figure 7). For MiSats, sequence variations were notably concentrated at specific positions, including those within the CENP-B box sequence. Notably, although 112-64-mers exhibit organizational differences compared to 120-mers, the divergence between different dimeric units within an array is relatively low, reaching up to 9.6%. This observation parallels the well-known characteristics of human alpha satellite higher-order repeats, where adjacent units display divergence while higher-order repeat units maintain higher homology. In contrast, MaSat and TLC Sat units displayed sequence variations distributed throughout their entire length (Now shown in Figure 4).

6) In the discussion, the authors state that "the extent of variations found in human centromere regions is higher compared to mice". How do the authors draw this conclusion? What kind of comparisons did they do and could the include such comparisons?

Authors: Unlike centromeres, where a single type of satellite array is present, human pericentric satellites have undergone extensive divergence, resulting in distinct satellite families. These include HSATI (comprising 17-bp and 25-bp repeat units), HSAT II (containing 10–80-bp repeat units), HSATIII (comprising 5-bp and 10-bp repeat units), beta satellites, and gamma satellites. Each HSAT family harbors unique DNA sequences, often displaying copy number variations in different cell lines. Additionally, human pericentric regions, particularly HSATI arrays, exhibit substantial structural rearrangements, including inversions. Furthermore, while centromeric α-satellites are mostly devoid of transposable elements (TEs), HSATI and HSATII repeat units are interspersed with ancient inactive TEs. This information, along with references, has now been added in the revised version (Introduction section, Page 3, second paragraph).

7) The authors should use a consistent color scheme for all figures showing TLC satellites and Telomeric satellites. Also, the colors used for the different repeats are in some cases hard to distinguish (e.g. two different shades of darker blue for two different repeats)

Authors: We have made the color scheme for TLC and telomeric satellite consistent. We have also changed the color scheme for major satellites to orange to distinguish them from blue-colored minor satellites.

8) For the centromere/telomere junctions, where is MiSat and why is it not shown? Is there a gap in the LRS data at the junction? Since the authors discuss the centromere/telomere junction, is unclear where the "centromere" part is supposed to be and what sequences are present at the transition.

Authors: We thank the reviewer for highlighting the need for more detailed information on centromere-telomere junctions in the original submission. Following the reviewer’s suggestion, we have included more analyses and information on TLC arrays at centromere telomere junctions. The data is now presented in the new Figure 7, and the text is added in the results section on pages 15-16 and the discussion section on page 20, third paragraph.

Arrays spanning telomere and centromere junctions contained four types of sequences (Figure 7B): TLC satellites, a short stretch of (TATACTCA)n simple repeats, 5’ truncated L1 element, and telomeric repeats (TTAGGG)n [67]. The 5’ truncated LINE- 1/L1 is a previously reported highly conserved element of centromere telomere junctions [42]. L1 is part of the Long interspersed nuclear elements (LINE) group of non-LTR retrotransposons that is highly abundant in almost all mammalian genomes [68]. A few TLC arrays contained LTR, such as RLTR17B_Mm. We detected TLC arrays spanning centromere-TLC as well as TLC-telomere junctions. On arrays spanning centromere-TLC junctions, TLC Sat units exhibited a high nucleotide variation (63-91.9%) throughout their respective satellite units (Figure 7B). On arrays spanning TLC Sats and telomeric repeats, while TLC arrays displayed high nucleotide variation (55.4-93.9%) within a given array, telomeric simple repeats were highly homogeneous (Figure 7B).

We also determined CENP-A, H3K9me3, and H3K27me chromatin enrichment on satellites at centromere-telomere junctions. MiSats present at these junctions exhibited a high level of CENP-A enrichment (~56-fold), while flanking TLC satellites showed a lower (up to ~5-fold) yet significant amount of CENP-A, implying CENP-A spreading from centromeres to TLC satellites (Figure 7C). Moreover, the 5’ truncated L1 elements at TLC-telomere junctions were depleted for CENP-A (Figure 7C). The L1 elements at the centromere-telomere junctions showed very low levels of H3K9me3 and H3K27me3 enrichment (1.12- and 1.17-fold, respectively), indicating weak or no heterochromatin at these sites (Figure 7C).

9) Figure 2 & 3: As mentioned before, sequence variation should be shown in a more quantitative way. Showing genome browser alignments is only partially informative.

Authors: We have included percent variation among satellite units within each array displayed in Figures 2, 3 and 7.

10) The alignment screenshots are positioned below individual repeats from what appear to be specific parts of the array. It's not clear what the arrows connecting the alignments and the repeats mean. Do the authors know that the variants shown below come from, say the left of the array? What is the basis for how are the variants grouped together for each array? This figure is quite confusing. As suggested in a previous comment, a more comprehensive and quantitative representation of the data would be preferred (e.g. deepTools)

Authors: We have arranged all the subunits within a specific array in the order they appear, spanning from the beginning to the end. Due to space constraints, we have presented the alignment solely for the top section of an array. Comprehensive sample alignments can be found in the supplementary figure 2 and 3. This information has been added to the figure legends. In addition, percent variation among satellite units within each array is now displayed in Figures 2, 3 and 7.

11) It it not clear what does the y-axis represent in 2E. It can be explained in figure legend.

Authors: The Y-axis represents the fraction of satellite units containing a specific type of CENP-B box variant in a given array. The keys to specific CENP-B variants are given alongside the graph.

We have now changed the fractions into percentages for simplicity. Therefore, in the new version of this figure, which is now Figure 4F, the Y-axis shows the % repeat units with intact or variant (indicated with specific colors) CENP-B boxes. Interestingly, among MiSat variants, the 112-64-dimeric MiSat variants exhibited a high percentage (67%) of intact CENP-B boxes, whereas the 112-mer MiSat variants displayed a low percentage (20%) of intact CENP-B boxes (Figure 4F). The density of the CENP-B box correlates with CENP-A enrichment as 112-64-dimeric arrays show higher CENP-A enrichment than 112-mer arrays (Figure 5A, 5B).

12) The authors mention in the text that some of the CENP-B box variants are predicted to not bind CENP-B. Which variants are not functional? What % of CENP-B boxes are predicted to be not functional for each array? This data would be interesting to correlate with abundance of CENP-A in each array to determine if CENP-B and CENP-A occupancies are positively correlated.

Authors: Specifically, within CENP-B boxes, positions 15-17, known to be conserved in functional CENP-B boxes (Reference 62-65), exhibited the highest sequence variations from the consensus. This point is covered in the new Figure 4A-B (showing sequence polymorphism at each position, including at the CENP-B box on MiSat repeat units) and Figure 4F (previously Figure 2E).

13) Figure 4 & supp fig 4: Again, TLC junction should be defined so readers can understand where the CENP-A and MiSat are present relative to the telomere.

Authors: We have defined TLC repeats in the introduction section (Introduction section, page 4, first paragraph)

14) Typo in Figure 6A 'Cchromatin organization'

Authors: Corrected

15) Figure 7 is not cited anywhere in the text.

Authors: We have moved the relevant schematic of centromeric and pericentric regions to Figure 1, as suggested by the reviewer. Considering that the previous Figure 7 only provided additional information already presented in the remaining figures, we removed it from the manuscript to make room for new Figures.

16) On Page 12, the authors state "At centromere and telomere junctions, TLC satellites were not enriched for CENP-A or H3K9me3 (Figure 4B, Supplementary Figure 4)." But the supplementary figure 4 shows a zoomed region on TLC with CENP- A and H3K9me3 peaks.

Authors: We thank the reviewer for pointing out this oversight. The text has been revised accordingly, and a new figure (Figure 7) has been added to address centromere-TLC junctions along with a detailed discussion on CENP-A enrichment on TLC satellites at centromere-telomere junctions (results section, pages 15-16 and discussion section, page 20, third paragraph). To enhance clarity, chromatin profiles for two full representative arrays spanning centromere-TLC and TLC-telomere junctions are now included (Supplementary Figure 4).

17) Something that I thought was missing in this manuscript is a discussion of the observation that CENP-A and H3K9me3 co-exist on MiSat. This is somewhat surprising based on fiber-IF data suggesting that H3K9me3 is not on the CENP-A region in human cells. Discussion of what we know about the two types of chromatin and how they are arranged in other system (with the caveat being that in some cases genomic data are lacking). Since the authors have data for nucleosome phasing, they can also speculate about why CENP-A and H3K9me3 are observed in the same positions, which could be explained by bulk-chromatin analysis afforded by ChIP-seq (e.g. in some cells CENP-A is present at a given position, in others H3K9me3, which would go against a sequence-specific binding pattern for CENP-A proposed by the authors in the discussion).

Authors: Excellent point. We have now performed additional analyses to address this important point. To identify the class of MaSat and MiSat arrays present at centromere- pericentromeric junctions, we isolated LRS reads containing both MiSats and MaSats and analyzed both sequence composition and chromatin organization on these arrays. The data is presented in the new Figure 7, and the text is added in the results section on pages 15-16 and the discussion section on page 20, third paragraph. We found that MiSats at centromere-pericentric junctions belonged to both the MiSat and MiSat variant classes, with sequence identities ranging from 85.3% to 100% (Figure 7A). Divergent MiSats present at centromere-pericentric junctions exhibited the lowest density of CENP-B boxes observed in MiSat arrays in this study, with arrays containing as little as 0.02% units with an intact CENP-B box (MiSat-MaSat arrays shown in Figure 7A, bottom panels). At these junctions, homogeneous MiSats, containing >50% units with an intact CENP-B box, displayed substantial CENP-A enrichment (~44-fold), while divergent MiSats, containing <50% units with an intact CENP-B box, exhibited lower yet significant levels (~7.5-fold) of CENP-A enrichment (Figure 7C). MiSats at centromere-pericentric junctions displayed ~1.7-fold H3K9me3 enrichment, suggesting minimal spreading from adjacent MaSat arrays (Figure 7C). Similarly, MaSats at centromere-pericentric junctions belonged to homogeneous and divergent classes. They demonstrated a notable amount of CENP-A enrichment (~2.7- fold), surpassing CENP-A enrichment (~1.5-fold) on randomly selected MaSat arrays from various classes, irrespective of their location on pericentric regions (Figure 6B, 7C). This suggests a localized spreading of CENP-A chromatin from centromeres to centromere-pericentric junctions (Figure 7C). MaSats at these junctions showed ~3.7- fold H3K9me3 enrichment, comparable to ~3.5-fold enrichment on homogeneous arrays containing only MaSats (Figure 7C).

Overall, our findings suggest that CENP-A nucleosomes spread from centromeres to pericentric regions and telomeres. CENP-A chromatin and H3K9me3 heterochromatin both occupy MiSats and MaSats spanning centromeric-pericentric junctions.

18) The authors should discuss phasing of CENP-A in other systems, when known.

Authors: A discussion about CENP-A phasing is now added in the introduction section (Introduction section, page 3, first paragraph).

19) The authors should include more details about the ChIP-seq methods rather than simply citing other papers.

Authors: We have now included a detailed ChIP protocol in the revised manuscript (Method section, Page 21, second paragraph)

Reviewer #2

The work presented by Packiaraj and Thakur on "DNA satellite and chromatin organisation at house mouse centromeres and pericentromeres" is well structured and presents innovative results that deserve publication, not least because these sequences have been neglected so many times. More recent data emphasize their importance for understanding the biology of chromosomes and the genome. Recent sequencing technologies bring new opportunities to study these complex and difficult sequences. The results of this work are innovative enough to be published in this important journal:

- Both centromeric minor satellites and pericentromeric major satellites exhibited sequence variations within and between arrays;

- Enrichment of CENP-A and H3K9me3 at both centromeric and pericentromeric regions differs on arrays containing different satellite variants;

- TEs at centromeric regions were not bound to CENP-A, indicating their absence from the functional centromeric domains.

- MaSat arrays contain H3K9me3 nucleosomes in a well-phased configuration, while MiSat arrays contain CENP-A nucleosomes that lack a phased configuration.

All this evidence may in fact reveal the structure needed to form a centromere, or rather a functional centromere.

Authors: We thank the reviewer for appreciating our work and for highlighting the current challenges in the field, thereby underscoring the relevance of this study.

**2^nd^ round**

**Reviewer 1**

The authors have addressed my questions and have improved the manuscript significantly. In the abstract:
" Next, using Chromatin immunoprecipitation sequencing (ChIP-seq), we found that the occupancy of CENP-A and H3K9me3 chromatin at centromeric and pericentric regions, respectively, is associated with increased sequence abundance and homogeneity at these regions." What do they mean by "sequence abundance"? Perhaps "enrichment" would be a clearer term
